# Medulloblastoma-associated mutations in the RNA helicase *DDX3X/DED1* cause defects in the translational response to TORC1 inhibition

**DOI:** 10.1016/j.jbc.2026.113580

**Published:** 2026-09-18

**Authors:** Akanksha Swarup, Ryan A. Kuhs, Vanessa U. Hardman, Kennedy L. Howard, Shradha Subbaraman, Timothy A. Bolger

**Affiliations:** Department of Biochemistry and Molecular Biology, University of Georgia, Athens, Georgia, USA

**Keywords:** RNA helicase, cancer, medulloblastoma, eukaryotic translation initiation, *Saccharomyces cerevisiae*, stress response, target of rapamycin

## Abstract

Medulloblastoma is the most common pediatric brain cancer, but current treatments are largely nonspecific, often causing developmental side effects. Genomic sequencing identified the RNA helicase *DDX3X* as one of the most frequently mutated genes in this cancer and a potential treatment target, yet its role in tumor progression remains elusive. Prior studies have indicated that the mutations cause specific defects in translation; however, both DDX3X and its yeast ortholog Ded1 have also been associated with cellular stress responses, suggesting that the contribution of the *DDX3X* mutations to medulloblastoma might result from defects in the translational response to stress. Building on our prior study that replicated the *DDX3X* mutations in yeast *DED1* (*ded1*-medulloblastoma-associated-mutants [*ded1-mam*]), we examined the mutants’ effects following Target of Rapamycin (TOR) pathway inactivation. First, we demonstrated that *ded1-mam* displayed substantial rapamycin-resistant growth compared to WT cells. In addition, similar to other *ded1* mutants, the *ded1-mam* had increased protein abundance of Ded1 and the translation factor eIF4G1 under TOR inactivation. Notably, these differences did not result in increased bulk translation following rapamycin; rather, the growth phenotypes appeared to be driven by translation of specific mRNAs. Reporter assays demonstrated enhanced translation of mRNA with an unstructured 5′ UTR in *ded1-mam* following TOR inhibition and a decrease in a structured reporter. Furthermore, known Ded1 target genes with relatively unstructured 5′ UTRs showed upregulated protein levels in rapamycin. We hypothesize that mutant DDX3X selectively upregulates translation of unstructured, progrowth transcripts while downregulating other structured transcripts, allowing tumor cells to bypass stress-induced growth controls and promoting medulloblastoma progression.

Medulloblastoma is the most common pediatric brain cancer, with around 400 new cases diagnosed in the United States each year (1, 2). Transcription profiling studies have classified medulloblastoma into four subtypes based on the affected signaling pathway: Wingless (WNT), Sonic Hedgehog, Group 3, and Group 4 (3). Current treatment strategies involve chemotherapy and radiation to the brain, and these procedures often result in long-term cognitive impairment and endocrine side effects (4). Therefore, more specific treatment strategies could help reduce the severe developmental effects of current broad-based treatments.

In recent years, deep sequencing studies have shown that the RNA helicase *DDX3X* is one of the most frequently mutated genes in medulloblastoma. These studies identified 42 different mutation sites in *DDX3X* in medulloblastoma patients, primarily associated with the WNT and Sonic Hedgehog subtypes, and all of these mutations are single amino acid substitutions or deletions (Fig. 1*A*) (4, 5, 6, 7, 8). DDX3X and its yeast ortholog Ded1 are members of the DEAD (Asp-Glu-Ala-Asp)-box protein family, which itself is part of Superfamily 2 (SF2) of ATP-dependent RNA helicases that are involved in ribonucleoprotein (RNP) remodeling (9, 10). Proteins that belong to the DEAD-box helicase family act in various roles in gene expression and RNA biology by unwinding double-stranded RNA and regulating RNP composition (9, 11, 12). The domain structure of DEAD-box proteins consists of a conserved helicase core region flanked by N- and C-terminal extensions that mediate regulatory and protein–protein interactions (Fig. 1*A*) (11).

**Figure 1:**
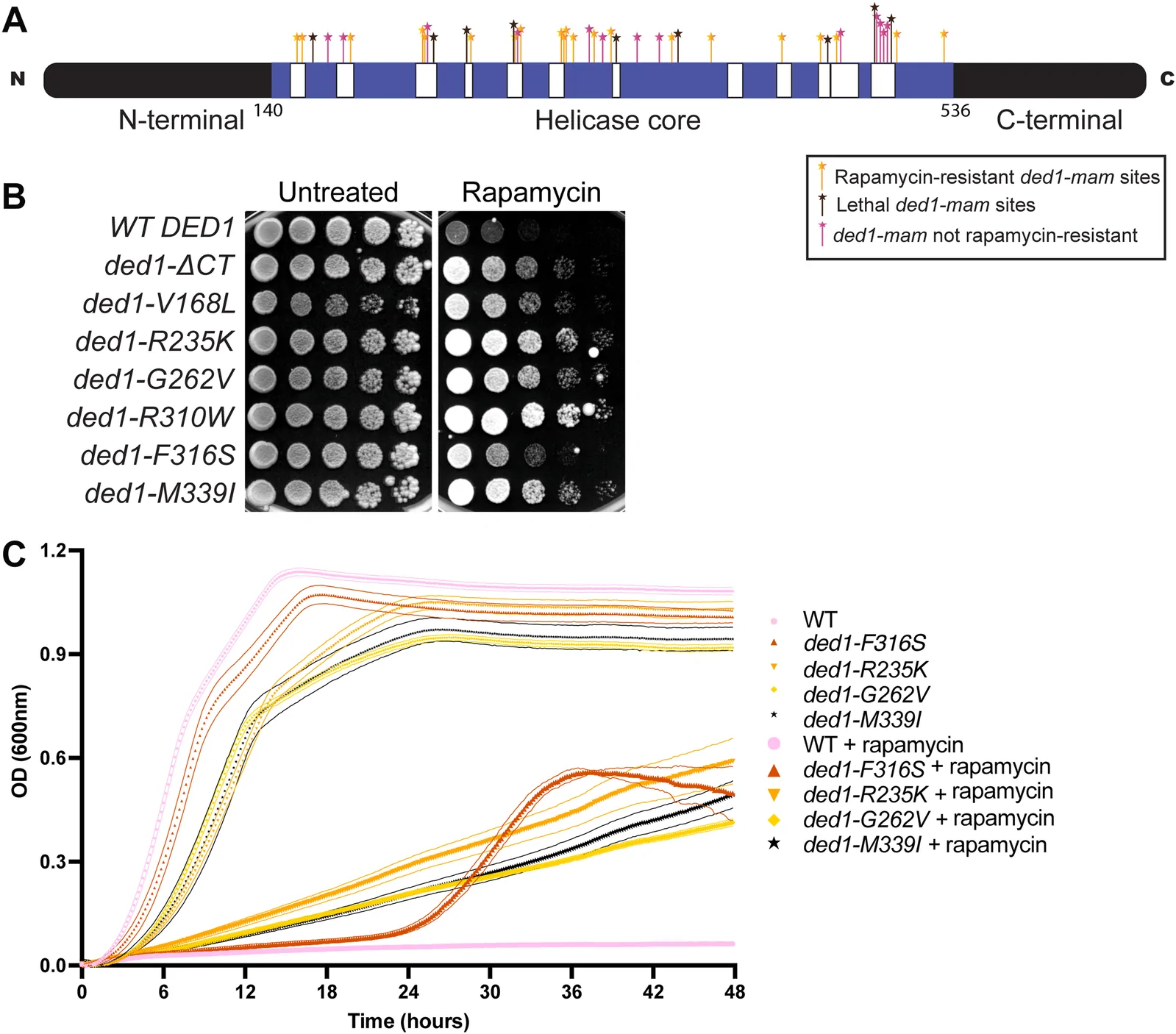
**Medulloblastoma-associated mutations in *DDX3X/DED1* show rapamycin-resistant growth.***A*, domain schematic of Ded1 showing the helicase domain (*blue*) with N- and C-terminal regions (*black*, amino acids 1–140 and 536–604, respectively) and conserved DEAD-box helicase motifs (*white boxes*). Medulloblastoma-associated mutation sites are shown as *stars* with lines indicating their location: rapamycin-resistant *ded1-mam* sites (*yellow*), *ded1-mam* sites that are not rapamycin-resistant (*pink*), and lethal *ded1-mam* sites (*black*). *B*, WT *DED1* cells and the indicated *ded1-mam* mutants were serially diluted onto nutrient-rich agar in the presence or absence of rapamycin (200 ng/ml) and incubated at 30 °C. Untreated colonies are shown after 2 days and rapamycin-treated colonies are shown after 5 days. *C*, WT *DED1, ded1-F316S, ded1-R235K, ded1-G262V,* and *ded1-M339I* cells were grown in liquid nutrient-rich media in the presence and absence of rapamycin, and cell concentration was measured *via* optical density at 10-min intervals for 48 h. For each strain/condition, the mean and SD for 3 replicates are shown. For rapamycin-treated cultures, measurements were fitted to an exponential growth equation. Growth parameters (k) for each *ded1-mam* strain were significantly different from WT cells (*p* < 0.0001). *ded1*-*mam*, *ded1*-medulloblastoma-associated-mutants.

DDX3X and Ded1 have conserved roles in eukaryotic translation initiation (10, 13). During initiation, translation factors and the 40S ribosomal subunit assemble on the mRNA to form the preinitiation complex (PIC) (14). DDX3X/Ded1 binds to components of the eukaryotic initiation factor 4F complex, which recognizes the 5′ end of target mRNA during PIC assembly, and Ded1 is believed to help facilitate this process (15, 16, 17). Following assembly, the PIC will scan the mRNA in the 5′ to 3′ direction, in search of the start codon. In a large subset of mRNAs, secondary structures in the 5′ UTR can impede this process, and DDX3X/Ded1’s most well-established role is to unwind these structures to facilitate start site scanning of these transcripts (18, 19, 20, 21, 22).

While DDX3X/Ded1’s role in translation initiation in nutrient-rich, progrowth conditions is well-studied, its role in other conditions, such as cellular stress, is less understood. In stress, global translation is normally repressed by multiple pathways, including Target of Rapamycin (TOR), a central mediator of cell growth and division (23).

In response to stressors such as nutrient depletion and DNA damage or treatment with rapamycin, TOR signaling is inhibited, which downregulates protein synthesis and cell division while increasing catabolic processes (24). This downregulation of translation involves, at least in part, eIF2α phosphorylation and induction of the integrated stress response (25, 26, 27). Our prior work, primarily utilizing a *ded1* mutant that deletes its C-terminal region (*ded1-ΔCT*), suggested that when the TOR pathway is inactivated, Ded1 acts as a translation repressor, helping the cell to halt growth and conserve energy during stress (28). Furthermore, this repression was at least partly mediated by the removal of the critical scaffolding protein eIF4G1 from translation complexes by Ded1, followed by the degradation of both proteins (28). However, DDX3X/Ded1’s role in stress appears to be complex. For example, in heat shock, it has been reported that Ded1 is repressed and unable to translate housekeeping genes with complex 5′ UTRs (29). These studies and others have suggested a model where DDX3X/Ded1 decreases growth and bulk translation while increasing translation of stress mRNAs in the initial stress response, followed by a role in the resumption of growth during stress adaptation and recovery (30). Taken together, these studies show that DDX3X/Ded1 plays an important role in the cellular stress response. Notably, stress responses are implicated in oncogenesis; tumor cells are frequently under stress, and thus, cancer drivers include alterations that allow cells to overcome stress-mediated growth inhibition (31).

In line with its involvement in brain cancer, DDX3X is also essential for normal brain development. *Ddx3x* is expressed throughout the developing brain in mouse embryos, and tissue-specific KOs result in impaired neural progenitor growth, decreased cortical generation, reduced neuronal differentiation, microcephaly, and apoptosis (32, 33). Furthermore, *Ddx3x* is critical for maintaining the balance between progenitors and neurons, regulating hindbrain patterning and development (34).

The 42 different mutations in *DDX3X* associated with medulloblastoma (see Fig. 1*A*) are spread across its conserved helicase core (35). Several studies have characterized small subsets of these mutations, and they have generally been found to impair translation. Protein levels of a known Ded1 target were decreased in a handful of medulloblastoma-associated DDX3X mutants in *Schizosaccharomyces pombe* (36), and overexpression of several DDX3X medulloblastoma mutant strains have displayed bulk translation inhibition (37, 38). However, many of the mutants do not appear to have defects in general translation, implying that the mutations do not primarily affect global translation but rather affect translation of specific genes (35).

Despite this prior research, a knowledge gap persists regarding the role of *DDX3X* mutations in cancer progression and the molecular mechanism behind this process. In particular, few studies have taken a broad approach to characterizing the spectrum of different mutations identified in medulloblastoma. We recently characterized all 42 mutants by leveraging the genetic tractability of yeast to generate mutants that replicate each of the 42 identified *DDX3X* mutations in its yeast ortholog, *DED1* (35). These *ded1*-medulloblastoma-associated-mutants (*ded1-mam*) displayed various growth and translation phenotypes. Still, the most common phenotype was a defect in translation of reporter mRNAs containing structured 5′ UTRs, suggesting that defective translation of this class of mRNAs may be responsible for the effect of the *DDX3X* mutations in medulloblastoma. However, this prior work did not consider the role of DDX3X/Ded1’s function in cellular stress.

Here, we examined the potential effect of the medulloblastoma-associated mutations on DDX3X/Ded1 stress function by again utilizing our *ded1-mam* lines. As shown below, we treated the *ded1-mam* mutants with the TOR inhibitor rapamycin to induce stress-like responses and observed their phenotypes. Most of the *ded1-mam* mutants demonstrated a rapamycin-resistant growth phenotype relative to WT, similar to our prior work with *ded1-ΔCT.* However, the mutants displayed bulk downregulation of translation under stress similar to WT cells, suggesting that more specific translation defects are responsible. We also observed that the mutants have increased persistence of Ded1 and eIF4G1 following TOR inactivation. Interestingly, we found that upon TOR inactivation, the medulloblastoma-associated mutants showed increased translation of a reporter mRNA with an unstructured 5′ UTR and decreased translation of a reporter with a structured 5′ UTR. Likewise, protein levels of selected progrowth, relatively unstructured targets of Ded1 were also increased in the mutant cells under stress. These results point to a prospective role of the *DDX3X/DED1* mutants in preferential unwinding and translation of certain mRNAs depending on 5′ UTR structure in combination with the availability of eIF4G1 to stimulate translation during stress conditions. We suggest that in medulloblastoma, *DDX3X*’s role in stress response is dysregulated by the identified mutations, allowing medulloblastoma cells to continue to proliferate in adverse conditions and contributing to tumor progression.

## Results

### Medulloblastoma-associated mutations in *DED1/DDX3X* improve growth following TOR inhibition

Prior studies have identified 42 different mutation sites in *DDX3X* associated with medulloblastoma progression, all of which are in the central helicase domains (Fig. 1*A*) (4, 5, 6, 7, 8). As described previously, we have taken advantage of the high degree of sequence homology in this region to construct yeast strains with the corresponding mutations in *DED1*, terming these *ded1 medulloblastoma-associated mutants* (*ded1-mam*) (35). Out of these 42 mutants, 9 of them were lethal (*ded1-V176KV, -Q240H, -G261V, -G284E, -T343P, -I374-, -A459-, -H485Y,* and *-R492S),* while the remaining *ded1-mam* mutants had varied growth and translation phenotypes consistent with established functions of Ded1/DDX3X (35). However, other than a moderate increase in stress granules (SGs) in some mutants, whether the functions of Ded1/DDX3X in stress responses are affected by these mutations is not yet clear (35, 38).

In demonstrating that Ded1 has a role in the translational stress response, Aryanpur *et al.* previously showed that some *ded1* mutants are resistant to growth inhibition caused by treatment with the TOR inhibitor rapamycin (28). Likewise, to begin to characterize the effects of the *ded1-mam* mutations during cellular stress, we screened the 33 viable *ded1-mam* mutants for changes in growth following TOR pathway inactivation *via* rapamycin (24). Plated on nutrient-rich media in the absence of rapamycin, all 33 viable *ded1-mam* strains showed at least some growth at 30 °C, and 16 displayed cold-sensitive growth defects at 25 °C (Table 1), consistent with our previous study (35). Representative growth assays are shown in Figure 1*B*, and a complete set with all tested strains is shown in Figures S1 and S2. Upon addition of rapamycin (200 ng/ml) to the media, growth of WT *DED1* cells was markedly inhibited at both 25 °C and 30 °C, as expected (Figs. 1*B* and S1). In contrast, *ded1-ΔCT*, a previously characterized mutant deleting the C-terminal domain (residues 536–604), grew better than the WT *DED1* strain in the presence of rapamycin at both temperatures, as shown previously (17, 28). Another previously characterized mutant, *ded1-120*, which is cold-sensitive and has impaired enzymatic activity, also grew better than WT cells in the presence of rapamycin (Figs. S1 and S2) (13, 28).

**Table 1:** Rapamycin-resistant phenotype and temperature sensitivity of *DDX3X/DED1* medulloblastoma-associated mutations

*DDX3X* mutation site	*DED1*	Amino acid substitution	Temperature- sensitive growth?	Rapamycin- resistant at 25 °C	Rapamycin- resistant at 30 °C
*DDX3X*			+++	−	−
-	*ded1-ΔCT*		+++	++	+++
-	*ded1-120*		−	+++	++
**204**	**166**	**T/A**	**++**	**+**	−
**206**	**168**	**V/L**	**+**	**++**	**+++**
214	V176	I/S,KV	ND	ND	ND
222	184	A/P	+++	−	−
227	189	G/V	+++	−	−
**231**	**193**	**T/A**	**+**	**+++**	**++**
**275**	**234**	**T/M**	**++**	**+**	−
**276**	**235**	**R/K, T**	**+**	**+++**	**+++**
277	236	E/Q	+++	−	−
281	240	Q/H	ND	ND	ND
302	261	G/V	ND	ND	ND
**303**	**262**	**G/V**	**+**	**+++**	**+++**
325	284	G/E,R	ND	ND	ND
**326**	**285**	**R/C**	**++**	**+++**	**++**
327	286	L/V	+++	−	−
**329**	**288**	**D/V**	**+++**	**+**	−
**351**	**310**	**R/W**	**+**	**+++**	**+++**
**353**	**312**	**L/F**	**++**	**+**	**+**
**354**	**313**	**D/H,V**	**+++**	**+**	−
**357**	**316**	**F/S**	**++**	**++**	**++**
368	326	D/V	+++	−	−
**370**	**329**	**M/R**	**+++**	**+**	−
376	335	R/C,S	+++	−	−
**380**	**339**	**M/I**	**+**	**+++**	**++**
384	343	T/P	ND	ND	ND
393	352	A/S,V	+++	−	−
405	364	V/L	+++	−	−
**412**	**371**	**S/F**	**+**	**+++**	**++**
415	374	I/-	ND	ND	ND
**434**	**393**	**L/-**	**+++**	**+**	**−**
**475**	**433**	**R/C**	**+++**	**+**	**+**
**496**	**454**	**V/M**	**+++**	**+**	**−**
501	459	A/-	ND	ND	ND
**504**	**462**	**G/V**	**+++**	**++**	**++**
506	464	D/Y	+++	−	−
527	485	H/Y	ND	ND	ND
528	486	R/H	++	−	−
530	488	G/A	++	−	−
532	490	T/M	+++	−	−
534	492	R/S,H,C	ND	ND	ND
**536**	**494**	**G/R**	**++**	**+**	**-**
**568**	**526**	**P/L**	**+++**	**-**	**+**

The identified *DDX3X* mutations organized by amino acid sequence with the orthologous change in the conserved Ded1 sequence. Growth assays of the *ded1-mam* strains were performed at 25 and 30 °C on rich media in the presence or absence of rapamycin (200 ng/ml). Growth phenotypes: no growth (−), slow growth (+), moderate growth (++), and high growth (+++). Rapamycin-resistant *ded1-mam* strains are in bold. ND = not determined, mutation is lethal at all temperatures.

Remarkably, we observed that most of the *ded1-mam* mutants (21 of 33) displayed a similar rapamycin-resistant phenotype (Table 1 and Figs. S1 and S2). Notably, many of the cold-sensitive growth phenotypes in the *ded1-mam* mutants were reversed compared to WT in the presence of rapamycin, although it should be noted that all strains grew more slowly in rapamycin than they did in rich media alone. There is also a clear correlation between the cold-sensitive growth defect in these strains in rich media and the extent of rapamycin-resistant growth (Fig. S3). To further support these results, we also conducted growth assays in liquid culture at 30 °C for 48 h in the presence or absence of rapamycin (Fig. 1*C*). The *ded1-mam* mutants showed at most only minor growth defects in untreated conditions. However, while WT *DED1* cells showed severe growth inhibition upon rapamycin treatment, each of the *ded1-mam* mutants tested grew significantly better, consistent with our results on solid media. This increased growth of *ded1-mam* compared to WT *DED1* strains in rapamycin suggests that these mutants have defects in responding to cellular stress, similar to the *ded1-ΔCT* and *ded1-120* strains (28). Furthermore, roughly two-thirds of *ded1-mam* mutants were at least partially rapamycin-resistant, suggesting that alterations in the function of Ded1/DDX3X in stress responses may be a common phenotype underlying the contributions of these mutations to medulloblastoma progression.

### Medulloblastoma-associated mutants of *DDX3X/DED1* show only minor effects on bulk translation following TOR inhibition

As discussed previously, DDX3X/Ded1 has established roles in both stimulating and repressing translation in different conditions (13, 17, 20, 28, 29, 30, 39). In particular, our prior research has shown that Ded1 downregulates bulk translation following TORC1 inhibition by rapamycin, and this downregulation is impaired in some *ded1* mutants (28). Therefore, we wanted to observe whether the *ded1-mam* mutants have defects in downregulating bulk translation in a similar fashion. We chose two of the *ded1-mam* mutants to test this: the *ded1-R310W* mutation is within motif II of the helicase domain and had a strong rapamycin-resistant growth phenotype, while the *ded1-F316S* mutation is not within a conserved helicase motif and showed a more moderate growth phenotype. First, we performed polyribosome profiling to determine the overall translation status of the two mutants relative to WT *DED1* during rapamycin treatment. After growing the mutant and WT cells at 30 °C and then treating for 40 min with rapamycin, profiles were generated by measuring the RNA concentration (Abs_254nm_) in cellular extracts subjected to sucrose density gradient centrifugation (Fig. 2, *A* and *F*). The polysome to 80S monosome (P/M) ratio was then calculated from the profile for each condition as a measure of translational activity (Fig. 2*G*) (35, 40, 41).

**Figure 2:**
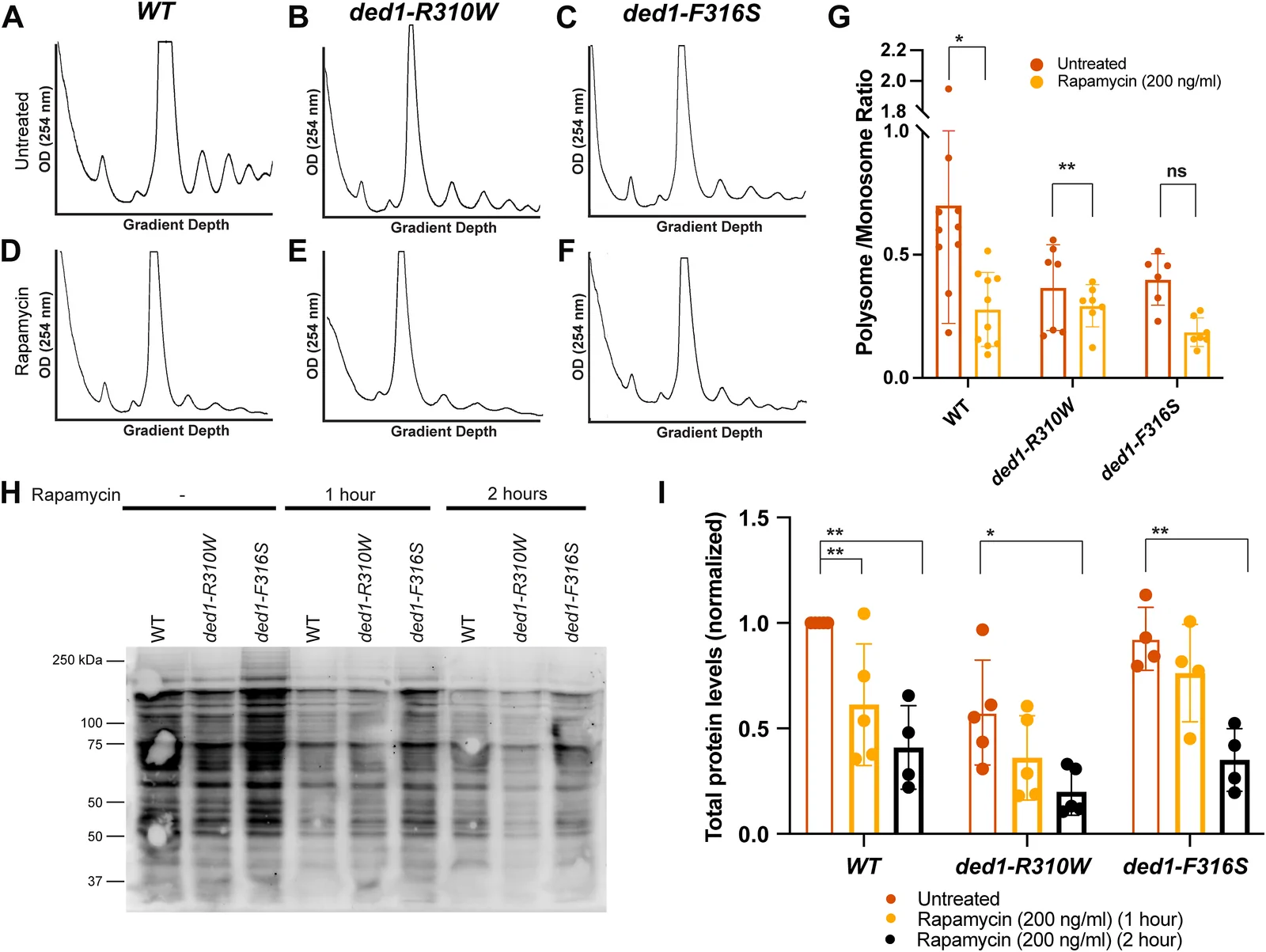
**Medulloblastoma-associated mutations of DDX3X/DED1 show only minor effects on bulk translation compared to WT following TOR inhibition.***A*, *B*, *C*, *D*, *E*, and *F*, polyribosome profiles of WT and the indicated *ded1-mam* mutants grown in the presence or absence of rapamycin for 40 min, following sucrose density centrifugation and OD_254nm_ analysis of cell lysates. *G*, the polysome/monosome (P/M) ratios were determined by comparing the sum of the areas of the polysome peaks with the area of the monosome peak. For each strain/condition, the mean P/M ratio and SD for 6 to 10 individual replicates are shown. *H*, WT *DED1* cells and the indicated *ded1-mam* mutants were treated with rapamycin (200 ng/ml) or not for 1 or 2 h, and simultaneously treated with puromycin (1 mM) for 1 h. Cell extracts were prepared, run on SDS-PAGE, and blotted with anti-puromycin to show newly-translated polypeptides. A representative image is shown. *I*, densitometry was performed on the total nascent polypeptide signal from each lane, normalized to levels in untreated WT *DED1* cells. For each strain/condition, the mean and SD for 5 individual replicates are shown. ∗*p* < 0.05, ∗∗*p* < 0.01, ns = not significant *versus* the untreated condition for each strain. *ded1*-*mam*, *ded1*-medulloblastoma-associated-mutants; TOR, Target of Rapamycin.

The polyribosome profile of WT cells grown in rich medium without rapamycin indicated robust translation activity, as expected (Fig. 2*A*). However, translation was somewhat reduced in both *ded1-mam* mutants in this condition with P/M ratios that were lower than that of WT *DED1*, although these differences were not significant (Fig. 2, *B*, *C* and *G*). This result is consistent with our prior study in which both *ded1-F316S* and *-R310W* showed moderately reduced P/M ratios after a shift to nonpermissive temperatures, suggesting a partial defect in bulk translation (35). After rapamycin treatment, bulk translation decreased substantially in WT *DED1* cells, as reflected in both the profiles and the P/M ratio, which decreased from 0.69 to 0.26 (Fig. 2, *D* and *G*), indicating general repression of translation in these conditions (42). Likewise, the bulk translational activity of both *ded1-F316S* and *-R310W* also declined in rapamycin (Fig. 2, *E* and *F*). The magnitude of the reductions in the mutants was not as great as in WT cells, resulting in P/M ratios (0.19 and 0.29) that were similar to the rapamycin-treated WT cells (Fig. 2*G*). Overall, there were no marked differences in translation in the *ded1-mam* mutants following TORC1 inhibition, unlike the relatively higher bulk translation observed previously in the *ded1-ΔCT* mutant in rapamycin (28).

To corroborate these findings, we also performed a puromycin incorporation assay (Fig. 2*H*). Puromycin was added to WT, *ded1-R310W* and *-F316S* cells for 1 h in the absence or presence of rapamycin for one or 2 h, and then cell extracts were blotted with anti-puromycin to detect the newly synthesized polypeptides. We observed that rapamycin treatment significantly reduced the puromycin signal in both WT and mutants in a time-dependent manner (Fig. 2*I*). Consistent with the polysome profiling, however, there were similar amounts of nascent polypeptides being produced in WT and *ded1-mam* cells for each condition. Differences between the two translation assays may reflect different sensitivities to changes in bulk translation. Regardless, since our results did not show significantly higher translation in the *ded1-mam* mutants compared to WT following rapamycin treatment, it is unlikely that the rapamycin-resistant growth phenotype observed in the *ded1-mam* mutants is due to a general increase in bulk translation. Rather, more specific changes in translation regulation may be responsible for this stress-resistant growth.

### Medulloblastoma-associated mutants of *DDX3X/DED1* demonstrate increased abundance of Ded1 and eIF4G1 upon TOR inactivation

Under normal conditions, it has been observed that Ded1 promotes 48S PIC formation and interacts with eIF4G1 *via* its C-terminus to support translation and cell growth (16, 17, 43). However, we had previously proposed a model where, under stress, WT Ded1 dissociates eIF4G1 from the PIC, leading to codegradation of both proteins and translation repression (28). In the *ded1-ΔCT* mutant, this remodeling of the PIC is reduced, and Ded1 and eIF4G1 degradation is slowed, resulting in increased translation and rapamycin-resistant growth. Further evidence suggested that the enzymatic activity of Ded1 is required for this remodeling (28). Therefore, we hypothesized that the *ded1-mam* mutations, which are found in the helicase domain and often showed moderately defective enzymatic activity in a previous study (35), may also cause a similar reduction in degradation of Ded1 and eIF4G1, resulting in increased protein abundance following rapamycin treatment.

To demonstrate this, we treated WT *DED1*, *ded1-ΔCT*, and six of the *ded1-mam* mutants (*ded1-V168L*, *-R235K*, *-G262V*, *-R310W*, *-F316S*, and *-M339I*) with rapamycin for 4 h. These strains were selected because they demonstrated rapamycin-resistant growth relative to the WT *DED1* strain in Figure 1, *B* and *C*. Cell extracts were prepared and run on SDS-PAGE gels to analyze levels of Ded1 and eIF4G1 in untreated and rapamycin-treated cells (Figs. 3*A* and S4). In WT *DED1* cells, we observed that Ded1 and eIF4G1 levels were higher in untreated conditions compared to after rapamycin treatment (Fig. 3*A*). Following rapamycin treatment, a sharp decrease in the levels of both Ded1 and eIF4G1 was seen, as expected (Fig. 3, *B* and *D*) (28). By contrast, in the *ded1-ΔCT* mutant, while there still appeared to be a decrease in Ded1 and eIF4G1 levels after rapamycin treatment, the residual protein levels were higher than in treated WT cells (Fig. 3, *A*, *C* and *E*), consistent with our prior studies (28).

**Figure 3:**
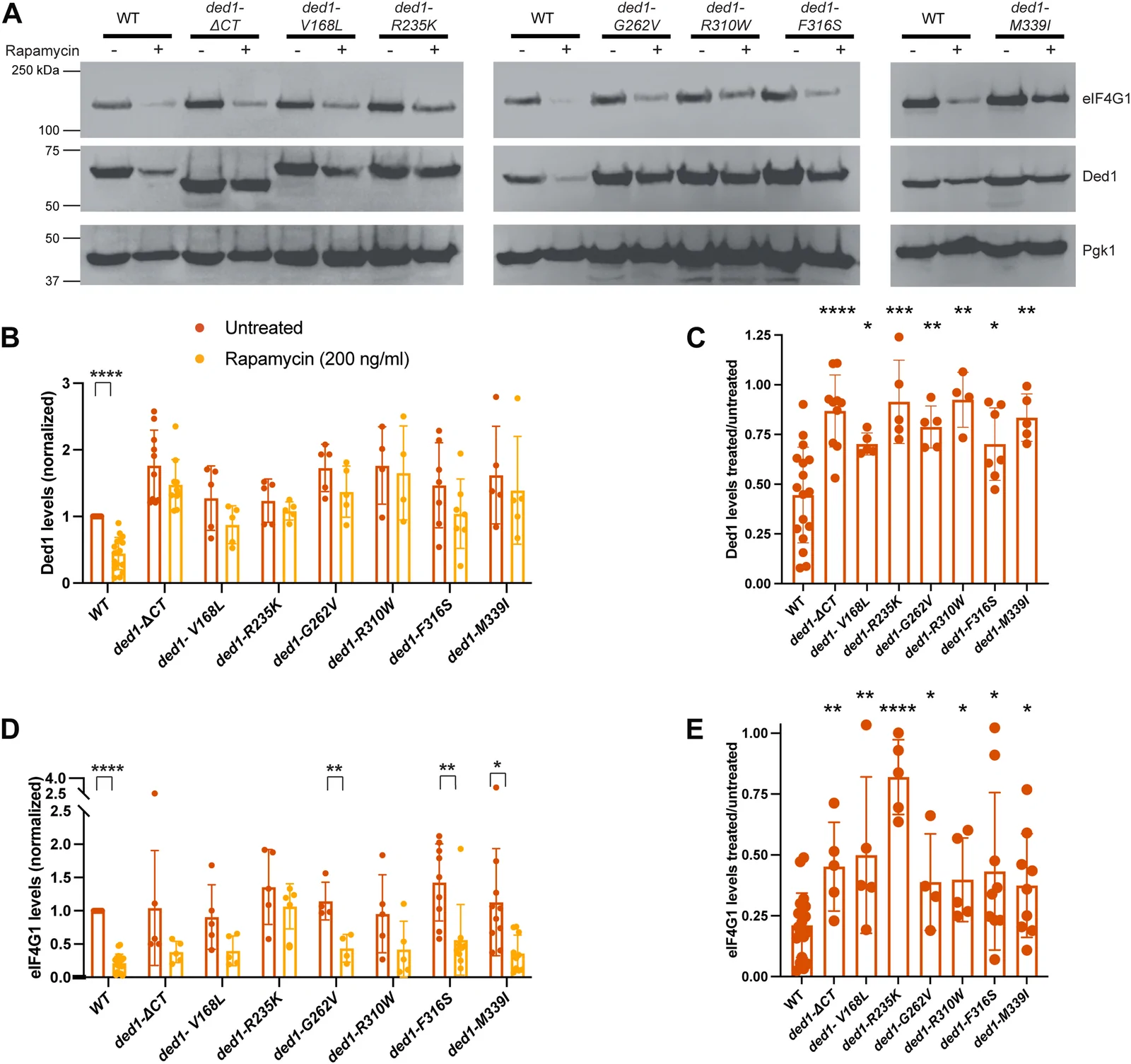
**Medulloblastoma-associated mutants of *DDX3X/DED1* display increased abundance of Ded1 and eIF4G1 upon TOR inactivation.***A*, protein extracts from WT *DED1* cells and the indicated *ded1-mam* mutants were treated with rapamycin (200 ng/ml) for 4 h or not, then cell extracts were run on SDS-PAGE and blotted using anti-Ded1, anti-eIF4G1 (Tif4631), and anti-Pgk1 (loading control). Representative images are shown. See Figure S4 for uncropped images. *B* and *D*, band densitometry was performed, and Ded1 and eIF4G1 protein levels, respectively, are shown, normalized to the Pgk1 loading control and levels in untreated WT *DED1* cells. ∗*p* < 0.05,*∗∗p* < 0.01, *∗∗∗∗p* < 0.0001 *versus* Ded1 levels in untreated cells. *C* and *E*, the ratios of Ded1 and eIF4G1, respectively, in treated *versus* untreated conditions are shown.*∗p* < 0.05, *∗∗p* < 0.01, ∗*∗∗p* < 0.001, *∗∗∗∗p* < 0.0001 *versus* WT *DED1*. For (*B*-*E*), the mean and SD for 4 to 19 individual replicates are shown. *ded1*-*mam*, *ded1*-medulloblastoma-associated-mutants; eIF4G1, eukaryotic initiation factor 4G1; TOR, target of rapamycin.

For the *ded1-mam* mutants, we observed phenotypes similar to *ded1-ΔCT*, wherein each of the mutants showed increased levels of Ded1 and eIF4G1 compared to WT cells following rapamycin treatment (Fig. 3, *A*–*E*). In each mutant, the reduction in Ded1 levels in treated compared to untreated conditions was roughly similar to the elevated levels observed in *ded1-ΔCT* (Fig. 3*C*). Likewise, for eIF4G1, the reduction in protein levels after treatment in the *ded1-mam* mutants was similar to that in *ded1-ΔCT* cells, except for *ded1-R235K*, which showed somewhat higher levels (Fig. 3*E*). Consistent with prior studies, only minor changes in the protein levels (<2-fold) were observed in untreated cells (35, 38). Thus, Ded1 and eIF4G1 show increased protein abundance upon rapamycin treatment in the *ded1* medulloblastoma-associated mutants, similar to our previous observations of *ded1-ΔCT* and consistent with reduced degradation in these conditions (28). These results suggest that the increased amount of Ded1 and eIF4G1 in these conditions is responsible for the increase in rapamycin-resistant growth in these mutants and may cause altered translation regulation.

### Medulloblastoma-associated mutants of *DDX3X/DED1* show opposing effects on translation in stress conditions depending on 5′ UTR structure

Because we did not observe substantial changes in bulk translation in the *ded1-mam* mutants during TOR inactivation, we hypothesized that the increased levels of eIF4G1 and Ded1 cause specific translation defects that are responsible for the mutants’ rapamycin-resistant growth. Both DDX3X and Ded1 have documented roles in facilitating start site scanning *via* unwinding secondary structures in 5′ UTRs of mRNAs (19, 20, 21, 22, ). To study how scanning through structured and unstructured 5′ UTRs is affected in *ded1-mam,* we obtained a previously published luciferase reporter with 5′ UTRs derived from *RPL41A* that either contain a stem-loop (ΔG_free_ = −3.7 kcal/mol) or are unstructured (21) (Fig. 4*A*). In our previous study, we examined the activity of this reporter in a subset of *ded1-mam* mutants under normal conditions, where the mutants showed consistently reduced translation of the reporter with the structured 5′ UTR compared to the one with the unstructured 5′ UTR, implying that the *ded1-mam* mutants were defective in unwinding of RNA structures in the 5′ UTR (35). These specific translation defects also correlated with the mutants’ cold-sensitive growth defects. However, how the *ded1-mam* mutants behave under stress conditions is unknown. To address this hypothesis, we transformed the reporter containing either a structured or unstructured 5′ UTR into WT *DED1* and the indicated *ded1-mam* mutants, left the cells untreated or treated with rapamycin for 4 h, and conducted luciferase assays. As shown in Figure 4*B*, luciferase activity from the structured reporter appeared lower than that from the unstructured reporter in both untreated and rapamycin-treated conditions, as expected (21, 35). Likewise, consistent with the general repression of translation following rapamycin treatment (44), both unstructured and structured reporter activity was generally decreased upon rapamycin treatment (Fig. 4*B*).

**Figure 4:**
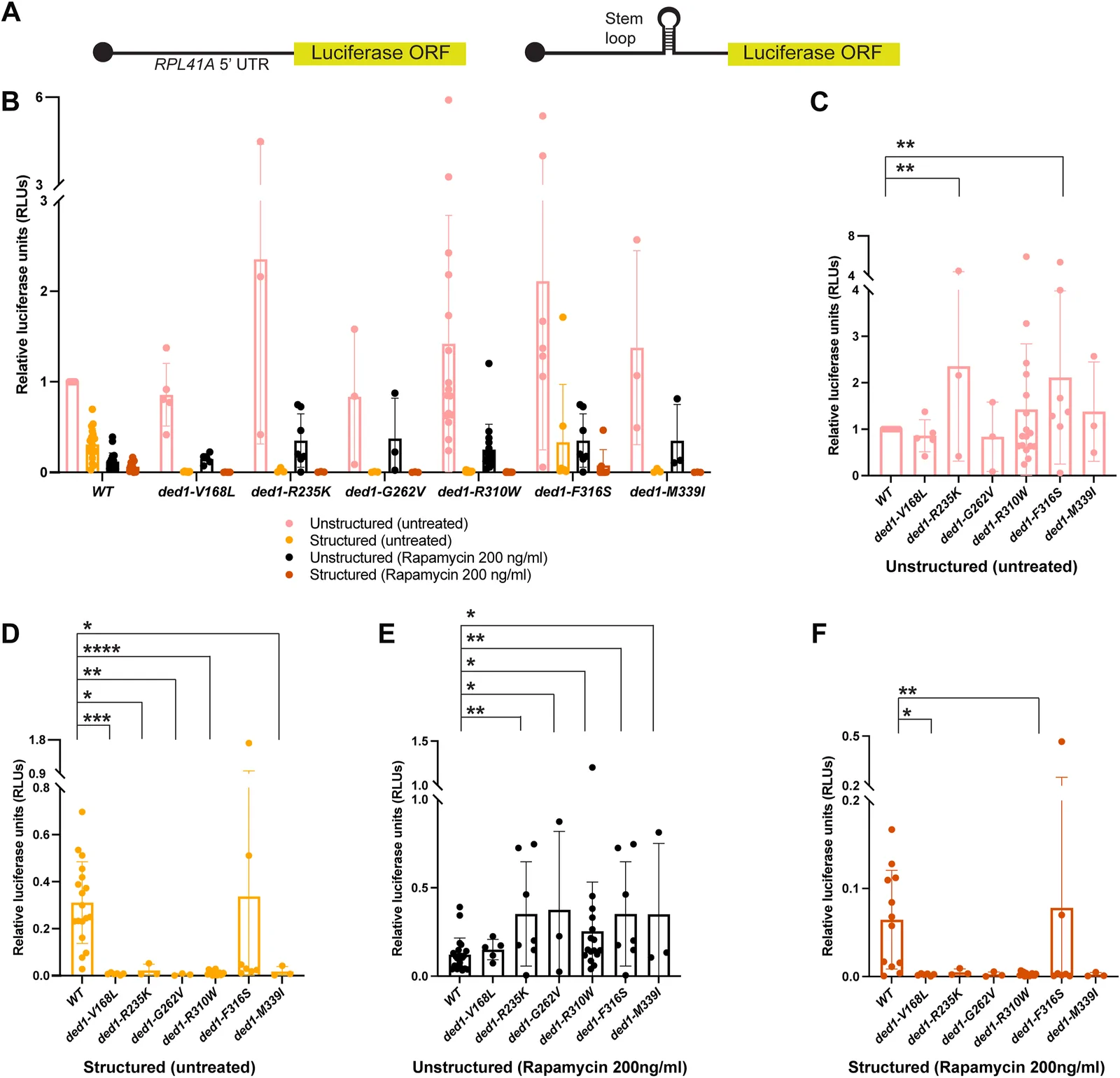
**Medulloblastoma-associated mutants of *DDX3X/DED1* show opposing effects on translation in stress conditions depending on 5′ UTR structure.***A*, schematic of the unstructured (*left*) and structured (*right*) 5′ UTR luciferase reporter. The 5′ UTRs are derived from that of *RPL41A*, lengthened to 91 nt, with a 3.7 kJ/mol stem loop inserted in the structured reporter. *B*, WT *DED1* cells and the indicated *ded1-mam* mutants containing either the unstructured or structured reporter were treated with rapamycin (200 ng/ml) or not for 4 h, and cell extracts were subjected to luciferase assays. The relative luciferase units (RLUs) are shown, normalized to untreated WT cells. These data were then replotted in *panels C-*F for ease of comparison between strains. *C*, RLUs are shown for WT *DED1* cells and the indicated *ded1-mam* mutants containing the unstructured 5′ UTR reporter in untreated conditions. *D*, RLUs are shown for WT *DED1* cells and the indicated *ded1-mam* mutants containing the structured 5′ UTR reporter in untreated conditions. *E*, RLUs are shown for WT *DED1* cells and the indicated *ded1-mam* mutants containing the unstructured 5′ UTR reporter, treated with 200 ng/ml rapamycin for 4 h. *F*, RLUs are shown for WT *DED1* cells and the indicated *ded1-mam* mutants containing the structured 5′ UTR reporter, treated with 200 ng/ml rapamycin for 4 h. For (*B*-*F*), the mean and SD for 3 to 16 individual replicates are shown. *∗p* < 0.05, *∗∗p* < 0.01, ∗∗∗*p* < 0.001, ∗∗∗∗*p* < 0.0001 *versus* WT *DED1* with the same reporter in the same conditions. *ded1*-*mam*, *ded1*-medulloblastoma-associated-mutants.

We then compared the activity in the *ded1-mam* mutants to that in WT *DED1* cells for each reporter and condition (Fig. 4, *C*–*F*). For the unstructured reporter in untreated conditions, the activity in *ded1-mam* cells was similar to WT *DED1*, although it was somewhat increased in *ded1-R235K* and *ded1-F316S* (Fig. 4*C*). As previously reported, however, the *ded1-mam* cells containing the reporter with a structured 5′ UTR showed significantly reduced luciferase activity compared to WT *DED1* under untreated conditions (Fig. 4*D*) (35), except in *ded1-F316S*, which showed highly variable results. In contrast, after rapamycin treatment, unstructured reporter activity was 2 to 3-fold higher in the *ded1-mam* mutants compared to WT *DED1* cells (Fig. 4*E*), except in *ded1-V168L*, which showed only a slight increase. Finally, consistent with the effect shown in untreated conditions, the activity from the structured reporter in *ded1-mam* cells also seemed to decrease compared to WT *DED1* under rapamycin treatment, although this difference was insignificant for some mutants, likely due to the very low activity in this condition (Fig. 4*F*). These results are especially intriguing since they imply a sharp contrast in the way that different classes of mRNAs are translated in *ded1-mam* mutants compared to WT cells. Specifically, a reporter mRNA with an unstructured 5′ UTR is significantly upregulated in the *ded1-mam* mutants relative to WT *DED1* under stress, and an mRNA with a structured 5′ UTR is downregulated in the *ded1-mam* cells. Moreover, these data suggest that the rapamycin-resistant growth of *ded1-mam* cells may be driven by the translation of mRNAs with relatively unstructured 5′ UTRs, while mRNAs with relatively structured 5′ UTRs may be downregulated.

### Medulloblastoma-associated mutants of *DDX3X/DED1* have increased protein levels of known *DED1* targets with unstructured 5′ UTRs following TOR inactivation

The luciferase assays shown in Figure 4 demonstrated that in conditions of TORC1 inhibition, *ded1-mam* cells had increased translation of the reporter with an unstructured 5′ UTR compared to WT *DED1*, and conversely, the mutants displayed decreased translation of a structured reporter in the same conditions. Furthermore, these results may suggest that the rapamycin-resistant phenotype of the *ded1-mam* cells is at least partially due to increased translation of mRNAs with relatively unstructured 5′ UTRs that encode proteins that support growth.

To validate this model, we tested whether previously identified mRNA targets of Ded1 with relatively unstructured 5′ UTRs showed increased protein levels in *ded1-mam* mutants under stress (Fig. S5). Drawing from previously published ribosome profiling (Ribo-seq) data, we arranged a list of mRNAs dependent on Ded1 in nonstress conditions by the structural complexity of their 5′ UTRs, as determined by Parallel Analysis of RNA Structure (PARS) score (21, 45). To then focus on genes linked to TOR inactivation, we retained only those that are annotated in the *Saccharomyces* Genome Database as displaying altered growth in rapamycin-treated cells when mutated. This yielded 102 genes that were arranged in ascending order by PARS score (Fig. 5*A*). Taking the relatively unstructured half of these genes (PARS scores ≤ PTC4), we then focused on genes that have been previously annotated as causing a rapamycin-dependent growth defect upon their deletion. This phenotype would imply that these genes contribute to growth and/or rapamycin resistance, and we further narrowed this list to genes previously linked to stress response pathways. From the resulting list of 17, we selected 3 “progrowth” genes to test for changes in the *ded1-mam* cells following rapamycin treatment: *EDE1*, *VMA5,* and *CST6*.

**Figure 5:**
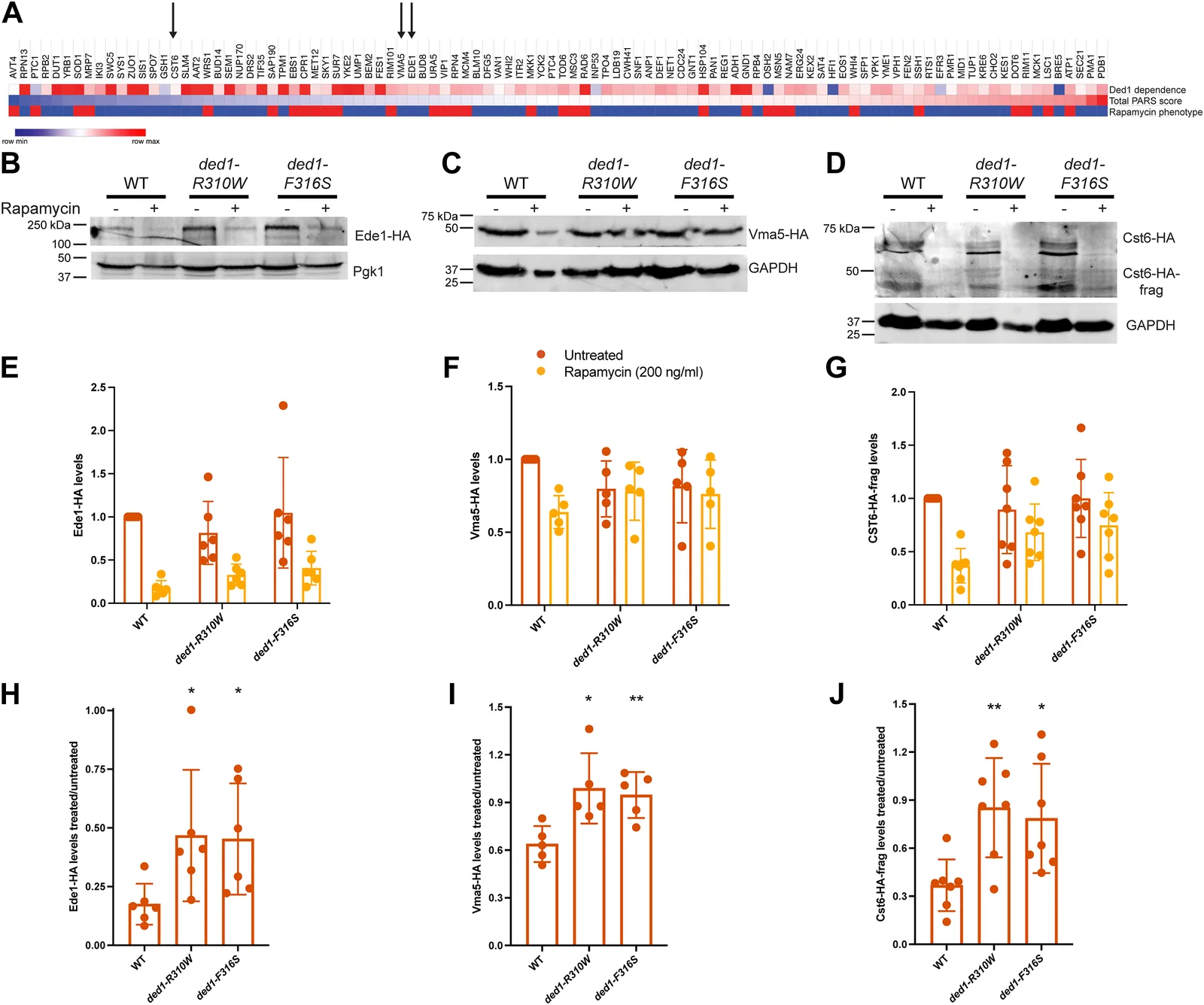
**Medulloblastoma-associated mutants of *DDX3X/DED1* have increased protein levels of known *DED1* targets with unstructured 5′ UTRs following TOR inactivation.***A*, heatmap showing the 102 Ded1-dependent genes that were annotated for rapamycin growth phenotypes, arranged in ascending order of their PARS score. Ded1 dependence is shown as the change in translation efficiency in a *ded1* temperature sensitive mutant where *red* is more sensitive to Ded1 perturbation. Total PARS score is shown where *blue* indicates less structure. Rapamycin-sensitive growth in a null mutant of the indicated gene is shown as *blue*, while rapamycin-resistance is shown in *red*. *EDE1*, *VMA5*, and *CST6*, three progrowth, rapamycin-resistant targets of Ded1 with relatively unstructured 5′ UTRs, are marked with *arrows*. *B*, *C*, and *D*, *EDE1*, *VMA5*, and *CST6* were tagged with 3xHA and their protein levels were examined in WT *DED1, ded1-R310W*, and *ded1-F316S*. Cells were treated with rapamycin (200 ng/ml), then protein extracts were run on SDS-PAGE and blotted using anti-HA with anti-Pgk1 and anti-GAPDH as loading controls. Representative images are shown. Note that two bands are present for Cst6 (at 65 kDa and 45 kDa), likely representing full-length and a fragmented version. See Figure S6 for uncropped images. *E*, *F*, and *G*, band densitometry was performed, and Ede1, Vma5, and Cst6 (45 kDa fragment) protein levels, respectively, are shown, normalized to levels in untreated WT *DED1*. *H*, *I*, and *J*, the ratios of Ede1, Vma5, and Cst6, levels, respectively, in treated *versus* untreated conditions are shown. *∗p* < 0.05, *∗∗p <* 0.01 *versus* the ratio for WT *DED1*. For (*E*-*J*), the means and SD for 5 to 7 individual replicates are shown. PARS, Parallel Analysis of RNA Structure; TOR, target of rapamycin.

To confirm our luciferase reporter results previously, we integrated an HA tag into these putative Ded1 target genes in WT *DED1* cells and two *ded1-mam* strains (*ded1-R310W* and *-F316S*). After treating these cells with rapamycin for 4 h, protein extracts were prepared and run on SDS-PAGE gels to analyze levels of the respective Ded1 targets in untreated and rapamycin-treated conditions. Consistent with our hypothesis, we observed that Ede1, Vma5, and Cst6 levels appeared to decrease in WT *DED1* cells upon rapamycin treatment (Figs. 5, *B*–*G* and S6). By contrast, these decreases in treated *versus* untreated cells were significantly reduced for each of the three proteins in the *ded1-R310W* and *ded1-F316S* mutants (Fig. 5, *H*–*J*). For Cst6, two separate bands were evident on the gel: one at its designated molecular weight (65 kDa) and a second at a lower molecular weight (45 kDa), likely a Cst6 fragment, that varied with the level of the 65 kDa species. Since the putative Cst6 fragment was often easier to visualize than the weak 65 kDa band, particularly following rapamycin treatment, the smaller band was used for densitometric analysis. Overall, these results imply that these progrowth genes that have relatively unstructured 5′ UTRs may be translated at an increased rate following TOR inhibition, which may contribute to increased cell growth in the *ded1-mam* mutants relative to WT *DED1*. However, *EDE1*, *VMA5*, *and CST6* are only examples of what are likely to be much larger classes of mRNAs translated differently in the mutants, so other mRNAs besides these are presumably contributing to these growth effects. Overall, these observations support a model where, in medulloblastoma, mutated DDX3X upregulates the expression of unstructured, progrowth genes, thus resulting in cancer progression and contributing to medulloblastoma pathology.

## Discussion

In this work, we have attempted to elucidate the molecular mechanism by which *DDX3X* mutations contribute to medulloblastoma progression. Using the genetically tractable yeast model, we introduced conserved medulloblastoma-associated mutations from mammalian *DDX3X* into the yeast homolog *DED1* (*ded1-mam*). In our prior studies with the *ded1-mam*, we discovered that they are not inactivating/null mutations and did not consistently reduce DDX3X/Ded1 function in different assays of activity (35). The extent of variability in growth and translation defects in the mutants suggested that they are not straightforward hypomorphic mutations, but they might share particular defects important in oncogenesis. We found that the growth defects in *ded1-mam* were best correlated with defects in their 5′ UTR unwinding activity, that is, these mutants had specific translation defects (35). However, this work focused on DDX3X/Ded1’s role under nutrient-rich, optimum conditions and did not take into account the role of DDX3X/Ded1 in stress responses.

In our current work, we expanded on this prior study and showed that roughly two-thirds of the mutants demonstrate rapamycin-resistant growth phenotypes (Figs. 1B, S1 and S2, and Table 1). These phenotypes are similar to both the C-terminal deletion mutant, *ded1-ΔCT*, as well as the enzymatically-deficient *ded1-120* mutant (28). Previously, we showed that many of the *ded1-mam* mutants had cold-sensitive growth defects in the absence of other stress, consistent with other studies and likely due to defects in helicase activity (35, 36, 46). Interestingly, here we observed a correlation between the two phenotypes (Fig. S3 and Table 1). Among the mildly temperature-sensitive mutants (marked as (+++) in Table 1), 8 out of 18 showed rapamycin-resistant growth; among the moderately temperature-sensitive mutants (++), 6 out of 8 were rapamycin-resistant; and among the extremely temperature-sensitive mutants (+), all 7 were rapamycin-resistant (Fig. S3). These results suggest that moderate defects in Ded1’s helicase activity might be behind this stress-resistance phenotype.

Numerous studies have established the critical role of DDX3X/Ded1 in translation (10, 13). Our prior work characterizing the *ded1-mam* mutants showed that while general, bulk translation was defective in some, this phenotype was highly inconsistent across a broad subset of the mutations (35). Here, we observed that relative to WT *DED1,* in the absence of rapamycin, the tested *ded1-mam* mutants show similar or moderately decreased bulk translation (depending on the assay), consistent with these previous results (Fig. 2). Under rapamycin treatment, their translation profiles likewise are not significantly different from WT *DED1*. Given these similarities in both polysome profiling and puromycinylation assays, it seems unlikely that changes in bulk translation, whether increased or decreased, are responsible for the *ded1-mam* growth phenotypes in rapamycin. Rather, it is more likely that specific changes in translation contribute to the rapamycin-resistance phenotype. Interestingly, these results are also consistent with the idea that growth and bulk translation do not always align perfectly, although translation is not completely absent in any of the conditions tested here.

Our previous work has also shown that under stress conditions, Ded1 triggers dissociation of eIF4G1 from mRNPs, resulting in the codegradation of both factors (28). However, in a *ded1* mutant that deletes the eIF4G1-interaction domain (*ded1-ΔCT*), this codegradation is reduced, resulting in higher Ded1 and eIF4G1 levels and facilitating continued translation. Likewise, here we also observed higher levels of Ded1 and eIF4G1 in *ded1-mam* strains relative to WT *DED1* following TOR inhibition (Fig. 3). Taken alone, this result suggests a mechanism similar to that observed in the *ded1-ΔCT* of decreased translation repression under stress. However, taken together with the translation assays in Figure 2, these results reinforce the implication that specific changes in translation are responsible for the rapamycin-resistance of *ded1-mam* and suggest that the levels of eIF4G1 and the mutated form of Ded1 may also contribute to these differences (see Fig. 6 below).

**Figure 6:**
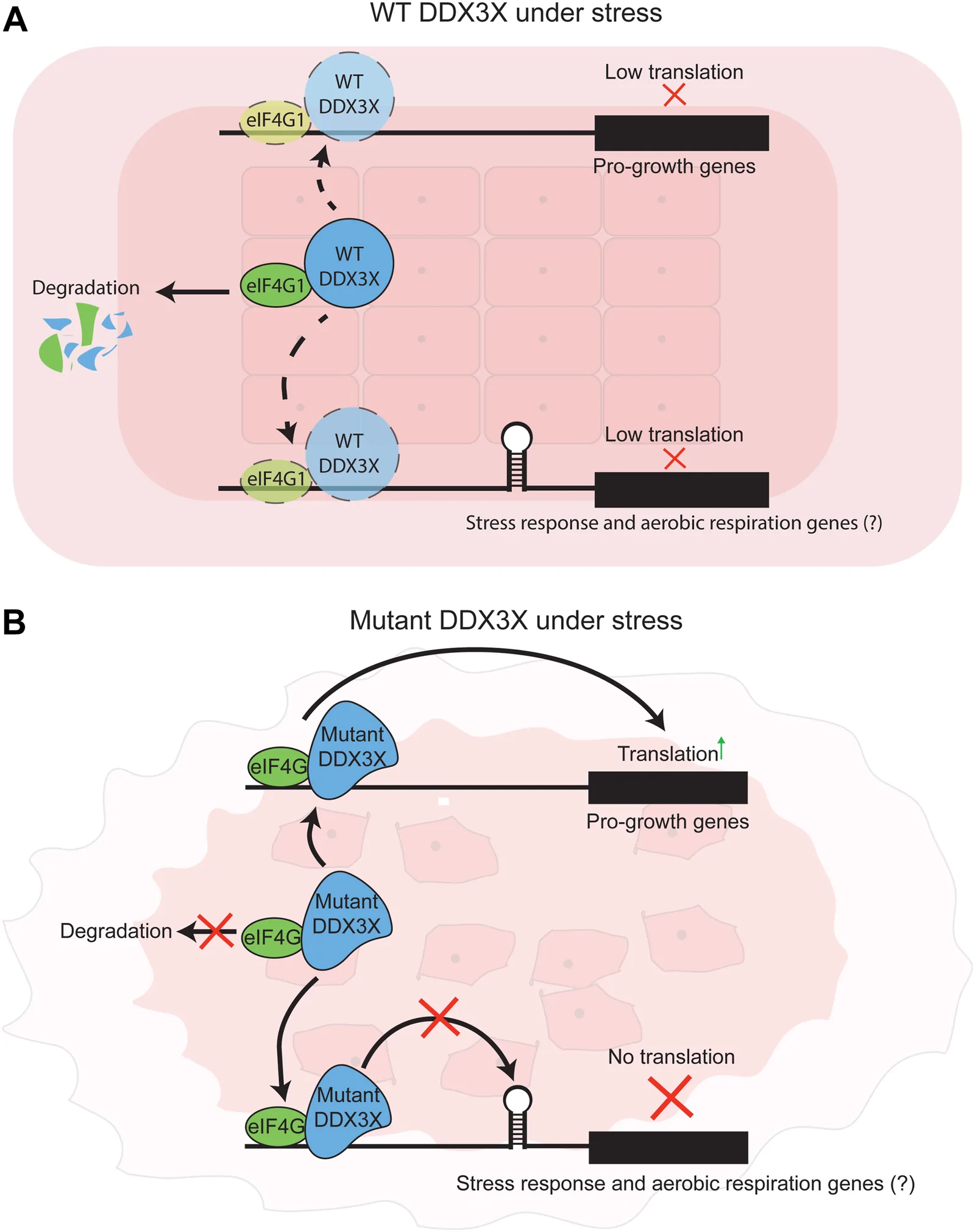
**Model for the role of DDX3X/Ded1 in medulloblastoma progression.***A*, normally, when cells encounter stress conditions, WT DDX3X will cause eIF4G1 dissociation from translation complexes, and both eIF4G1 and DDX3X will subsequently be degraded. The low levels of both proteins will downregulate translation for both progrowth mRNAs with unstructured 5′ UTRs and mRNAs with structured 5′ UTRs, which may include inhibitors of the stress response and/or aerobic respiration genes. Some mRNAs with more complex regulatory mechanisms will presumably still be translated (not pictured). *B*, however, in *ddx3x-mam* mutant cells, the aberrant DDX3X protein does not degrade eIF4G1, allowing continued translation of unstructured, progrowth genes and helping tumor cells bypass stress-induced growth inhibition. Conversely, the mutant DDX3X is not able to support translation of mRNAs with structured 5′ UTRs, possibly resulting in a partial stress response and changes in energy metabolism in the tumor. eIF4G1, eukaryotic initiation factor 4G1.

Prior research has also shown that DDX3X/Ded1’s function in unwinding secondary structures in the 5′ UTR for facilitating start site scanning is affected by these medulloblastoma mutations (35). Therefore, we hypothesized that this deficiency contributes to the rapamycin-resistant phenotypes in the *ded1-mam* cells. As shown in Figure 4, *ded1-mam* mutants translated the structured 5′ UTR reporter at a decreased rate relative to WT, regardless of the presence of rapamycin. This result extends our prior study, suggesting that the *ded1-mam* have difficulty unwinding structures in 5′ UTRs under any conditions. However, for the unstructured 5′ UTR reporter, we saw that they were translated in *ded1-mam* at a rate higher than WT *DED1* when treated with rapamycin. These observations suggest that the rapamycin-resistant growth of the *ded1-mam* cells is due to the increased translation of mRNAs with relatively unstructured 5′ UTRs that promote growth and would normally be repressed under stress conditions. Conversely, translation of mRNAs with relatively structured 5′ UTRs that encode stress-response or growth-inhibitory proteins may be decreased in the *ded1-mam* mutants, thus also contributing to the increased growth phenotypes in rapamycin.

As discussed previously, our data suggest that the *DDX3X/DED1* medulloblastoma-associated mutations affect the translatome in a complex manner, probably *via* the effects of DDX3X’s ability to unwind 5′ UTR structures. This model is also consistent with that of Oh *et al.*, who likewise proposed that there would be specific subsets of genes that would be selectively translated during stress conditions in the medulloblastoma-associated *DDX3X-R543H* mutant (37). To test whether our luciferase reporter findings can further inform this model, we identified genes related to growth and stress responses from previously identified Ded1-targeted mRNAs (21). Following TOR inhibition, we observed that the protein levels of progrowth genes that had relatively unstructured 5′ UTRs showed significantly higher abundance in *ded1-mam* relative to WT cells, implying that these mRNAs have increased translation in the mutants compared to WT cells in these conditions, consistent with the previous model (Fig. 5). However, we were not able to conclusively confirm the parallel hypothesis for candidate mRNAs with structured 5′ UTRs in preliminary assays, so more extensive testing may be required to investigate this possibility.

Taken together with previous studies, our results suggest a model wherein Ded1/DDX3X normally promotes translation repression of progrowth genes during stress conditions, at least partially through dissociation and degradation of eIF4G1, in order to support the redirection of resources from cell growth to cell survival (Fig. 6, top). However, medulloblastoma-associated mutants of Ded1 are not able to repress translation, and eIF4G1 is maintained at higher levels, promoting continued growth even during rapamycin treatment (Fig. 6, bottom). Extrapolating to cancer, mutation of *DDX3X* would likewise promote the translation of progrowth mRNAs (with unstructured 5′ UTRs), particularly in the interior of a tumor where cancer cells have limited nutrients and other stress conditions are present (47). Correspondingly, other subsets of mRNAs, such as inhibitors of the stress response and/or enzymes involved in aerobic respiration, that have structured 5′ UTRs will be further downregulated in cells with mutant Ded1/DDX3X due to defects in its helicase activity. In medulloblastoma, this would result in the upregulation of stress-response genes (antioxidants, chaperones, *etc.*) and/or metabolic changes in the tumor, respectively. Given the identified roles of DDX3X in neuron generation and differentiation (32, 33, 34), mRNAs involved in these processes may be regulated similarly in a *DDX3X* mutant to promote medulloblastoma progression.

This model suggests that *DDX3X/DED1* does not fit neatly into the categories of tumor suppressor or proto-oncogene, displaying properties of both depending on the target mRNA. These selective effects, while broadly consistent with other studies of the medulloblastoma mutants, have not been widely demonstrated (35, 37). As mentioned previously, Oh *et al.* proposed selective effects during stress conditions, specifically oxidative stress, although they observed only moderate defects in one mutant (37). Another study used a conditional KO model of *DDX3X* to suggest that it acts as a tumor suppressor by moderating the effects of upregulated Wnt signaling, which include cell death, cell cycle arrest, and “oncogenic stress” (34). However, a *ddx3x* KO may not fully recapitulate the effects of what appear to be complex hypomorphic mutations in medulloblastoma. Furthermore, only RNA levels were examined rather than translation itself, so some caution is needed in interpreting these results. Our data extend these results by both suggesting a potential mechanism and by proposing an additional layer of regulation. Along these lines, Valentin-Vega *et al.*, as well as others, observed that some of the medulloblastoma mutants exhibited increased SG formation (35, 37, 38). Although increased SGs are linked to cancer, their role remains somewhat unclear (48). In this study, we note that rapamycin does not induce SG formation in yeast, so the *ded1-mam* phenotypes we have observed presumably are not due to SGs.

This study defines the role of *ded1-mam* mutants in mediating the cellular stress response and provides new mechanistic insight into the conserved function of DDX3X/Ded1 in cancer. Our findings suggest that DDX3X/Ded1 selectively unwinds and promotes the translation of specific mRNAs based on 5′UTR structure, a process modulated by eIF4G1 availability under stress conditions. Dysregulation of this mechanism in *DDX3X/DED1* mutants enables medulloblastoma cells to sustain translation and proliferate under adverse conditions, thereby contributing to tumor progression. Given these striking phenotypes in yeast cells, future studies should include validating these stress-adaptive phenotypes in human cancer cells, possibly by using CRISPR/Cas9 to generate the appropriate *ddx3x* mutants in neurons or neuronally-derived cell lines. In addition, ribosome profiling of these mutants and verification of *DDX3X*’s translational targets in such a model would be critical to furthering our knowledge of DDX3X’s precise role in tumor progression. Such insights could help us identify therapeutic targets for medulloblastoma and inform more improved strategies for targeted treatment.

## Experimental procedures

### Strain and plasmid construction

Yeast strains and plasmids used are listed in Tables S1 and S2. The *ded1-mam* strains were generated as described previously (35). Ded1 target strains (TBY258, 262, 266) were generated by integrating a C-terminal 3 x-HA tag marked with *HIS3* at the respective locus (*CST6, VMA5*, *EDE1*) into the yeast strain SWY4093 (*ded1::KAN + pDED1/URA3/CEN*). The *DED1* and *ded1-*mam plasmids were then shuffled into these parental strains.

### Growth assays

Yeast growth assays were performed by serial dilution as previously described on rich media (YPD) in the presence or absence of 200 ng/ml rapamycin (28). Images were taken after 1 to 2 days for the YPD plates at 30 °C, 2 to 3 days for YPD plates at 25 °C, and 6 to 8 days for plates supplemented with rapamycin. Growth phenotypes of the strains were then qualitatively assessed. For denoting temperature-sensitive growth, strains with minimal growth at 25 °C were labeled as (−), slow growth as (+), moderate growth as (++), and growth equivalent to WT *DED1* as (+++). For rapamycin-resistant growth at 25 °C and 30 °C, the growth of *ded1-mam* was compared to the growth of WT *DED1* on the same plate. This difference was also compared to the mutant’s growth on untreated media (*e.g.*, a mutant that grew more slowly on untreated plates was scored as rapamycin-resistant for the same growth level on rapamycin.) Growth on rapamycin equivalent to WT *DED1* was labeled as (−), slightly more growth as (+), moderate growth as (++), and the best growing mutants were labeled as (+++).

For liquid growth assays, log-phase cells were diluted to an absorbance (OD_600nm_) of around 0.02 in liquid YPD in the presence or absence of rapamycin in triplicate in 100-well plates. Cultures were then incubated in a Bioscreen C° Pro at 30 °C, shaken every 10 min, and optical density measurements were taken every 10 min for 48 h. From this data, graphs were generated showing the mean and SD for each triplicate at each time point. For rapamycin-treated cultures, data were fitted to exponential growth equations and compared *via* the extra sum-of-squares F test in GraphPad Prism.

### Western blotting

Blotting for protein levels of Ded, eIF4G1 (Tif4631), and Ded1 targets was performed as previously described (43). Cells were grown to early-log phase and then subjected to rapamycin treatment (200 ng/ml) for 4 h. Crude cell lysates were prepared by lysing the cells in 1.85 M NaOH and 7.4% beta-mercaptoethanol, followed by trichloroacetic acid precipitation. Proteins were separated by SDS-PAGE and probed with antibodies specific against Ded1 (49), eIF4G1 (28), and HA Tag (12CA5, Invitrogen, #26183) for Vma5, Ede1, and Cst6. Pgk1 (22C5D8, Abcam, #ab113687) and GAPDH (Invitrogen, #MA5-15738) were used as loading controls. Fluorescent secondary antibodies (IRDye 680RD Goat Anti-Rabbit IgG and IRDye 800CW Goat anti-Mouse IgG) were used to visualize bands on an Odyssey M imager (LI-COR Biosystems). For densitometry analysis, protein band intensity was measured with ImageJ/Fiji software. Ded1 and eIF4G1 levels in untreated samples were normalized to Pgk1 in the respective untreated sample, and Ded1 and eIF4G1 levels in rapamycin-treated samples were normalized to Pgk1 in the respective treated sample. For Ede1 and Vma5, samples were analyzed similarly and normalized to Pgk1 and GAPDH, respectively. For Cst6, two bands were observed at 65 kDa (its designated molecular weight) and at 45 kDa (likely a fragment). The 45 kDa band was used for densitometry after normalizing to GAPDH. For calculating the ratio of treated to untreated protein levels (Figs. 3, *C* and *E* and 5, *H*–*J*), values for rapamycin-treated samples were divided by the values for untreated samples in the same replicate and then averaged across replicates. Statistical significance was calculated using unpaired, two-tailed Student’s *t* tests.

### Bulk translation assays

Polysome profiles were generated essentially as described previously (43). Briefly, WT *DED1, ded1-R310W,* and *ded1-F316S* were grown to midlog phase, and 200 ng/ml of rapamycin was added for 40 min. Ribosomes were stalled on mRNAs *via* the addition of cycloheximide (100 μg/ml), and then cells were lysed using a bead beater (2× for 2.5 min). Lysates were loaded onto a 7 to 47% sucrose density gradient and centrifuged for 2.5 h at 38,000 rpm. RNA concentration of the samples was then monitored *via* absorbance at 254 nm during continuous fractionation. Polysome/monosome ratios were calculated by comparing the sum of the areas under the polysome peaks to the area under the 80S monosome peak in ImageJ/Fiji. Statistical significance was determined *via* Mann-Whitney nonparametric tests in GraphPad Prism.

Puromycin incorporation assays were performed by growing cells to early-log phase and then subjecting them to rapamycin treatment (200 ng/ml) for 1 and 2 h, and simultaneous puromycin treatment (1 mM) for 1 h. Crude cell lysates were prepared by lysing equal numbers of input cells in 1.85 M NaOH and 7.4% beta-mercaptoethanol, followed by trichloroacetic acid precipitation. Proteins were separated by SDS-PAGE and probed with antibodies specific against puromycin (Merck, #MABE343). Fluorescent secondary antibody (IRDye 800CW Goat anti-Mouse IgG) was used to visualize bands on an Odyssey M imager (LI-COR Biosystems). For densitometry analysis, total signal intensity in each lane was measured with ImageJ/Fiji software, and values were normalized to the untreated WT signal on each blot. Statistical significance was calculated using unpaired, two-tailed Student’s *t* tests.

### Translation scanning assays

Scanning assays to study the translation of mRNAs with different 5′ UTR sequences were carried out essentially as described (21). WT *DED1* and the indicated *ded1-mam* mutant cells were transformed with an unstructured *RPL41A* 5′ UTR-firefly luciferase reporter (pFJZ342) or a distal-stem-loop-containing version (ΔG = −3.7 kcal/mol, pFJZ623). Transformants were cultured in triplicate in early log phase and then treated with rapamycin (200 ng/ml) for 4 h. Cell lysates were generated, and luciferase assays were performed with standard luciferin reagent (Promega) on a Glomax 20/20 luminometer (Promega) as previously described (35). Triplicates were normalized to their respective cell concentrations and averaged together for each biological replicate, then divided by the average light units for the unstructured reporter in untreated WT cells in each assay. For ease of comparing activity in WT and mutant cells for each reporter and condition, Figure 4, *C*–*F* replot the data shown in Figure 4*B*. Statistical significance was determined using unpaired, two-tailed Student’s *t* tests comparing the value in WT *DED1* to the *ded1-mam* mutant in the respective reporter and condition.

### Selection of Ded1-dependent mRNAs

To identify unstructured mRNAs that are dependent on Ded1 activity, previously published ribosome sequencing data were used, and PARS scores, as determined by Kertesz *et al.*, were used as a proxy for structural complexity of the 5′ UTR (21, 45). From a master list of 814 genes that were Ded1-dependent, 457 of them had designated PARS scores (Fig. S4). To narrow down this list further to select for genes involved in TOR inactivation, the Alliance Mine tool on the *Saccharomyces* Genome Database was used to select for genes with a growth phenotype change in the presence of sirolimus/rapamycin. These filters gave 102 (out of the 457) genes annotated for increased or decreased growth in rapamycin-treated cells when mutated. These 102 genes were arranged in ascending order of their PARS score (see Fig. 5*A*). The first half of these genes (PARS scores ≤ *PTC4*) were designated as relatively unstructured. Genes previously determined to be positively regulated by Ded1 (*i.e.*, they showed a negative change in translation efficiency for the *ded1-ts* mutant compared to the WT control) were selected (21), narrowing the list to 32 genes. Genes wherein the null mutant was rapamycin-sensitive (the expected phenotype, given that increased translation of these genes in *ded1-mam* was predicted) left 17 genes. From this list, 3 stress-related and “progrowth” genes (based on their phenotypes and known functions), *CST6*, *VMA5*, and *EDE1*, were selected for further analysis.

## Data availability

All data are contained within the article or supplementary information. Original data will be shared upon request of the corresponding author.

## Supporting information

This article contains supporting information (50, 51)

## Conflict of interest

The authors declare that they have no conflicts of interest with the contents of this article.
