# Supplementary material for: Medulloblastoma-associated mutations in the RNA helicase DDX3X/DED1 cause defects in the translational response to TORC1 inhibition
Source: J Biol Chem. 2026 Sep 18;302(11):113580. doi: 10.1016/j.jbc.2026.113580 (PMC13693076; doi:10.1016/j.jbc.2026.113580)
Supplement: Supplementary file 1 [file mmc1.pdf]

Supporting information

1. Supplementary table S1: Yeast strains used in this study
2. Supplementary table S2: Plasmids used in this study
3. Figure S1: Growth and rapamycin-sensitivity of DDX3X/DED1 medulloblastoma-associated mutations at 25°C.
4. Figure S2: Growth and rapamycin-sensitivity of DDX3X/DED1 medulloblastoma-associated mutations at 30°C.
5. Figure S3: Correlation between growth phenotypes and rapamycin-resistance of *ded1-mam* cells.
6. Figure S4: Original, uncropped western blots of Fig 3A.
7. Figure S5: Selection of Ded1-dependent genes with relatively unstructured 5' UTRs.
8. Figure S6: Original, uncropped western blots of Fig 5B-D

Supplementary table S1: Yeast strains used in this study

Strain name	Genotype	Source
W303	*MATα ade2-1 ura3-1 his3-11,15 leu2-3,112 trp1-1 can1-100*	(50)
SWY4093	*MATα ded1::KANr ade2-1 ura3-1 his3-11,15 leu2-3,112 trp1-1 can1-100 +pCEN/URA3/DED1*	(49)
TBY52	*MATα ded1::KAN ade2-1 ura3-1 his3-11,15 leu2-3,112 trp1-1 can1-100 +pCEN/LEU2/DED1 (pSW3619)*	(28)
TBY 121	*MATαded1::KAN ade2-1 ura3-1 his3-11,15 leu2-3,112 trp1-1 can1-100 +pCEN/LEU2/ded1-ΔCT*	(28)
SWY4277	MATαded1::KAN ade2-1 ura3-1 his3-11,15 leu2-3,112 trp1-1 can1-100 +pCEN/LEU2/*ded1-120*	(49)
TBY 258	*MATαded1::KAN CST6:3XHA:HIS3 ade2-1 ura3-1 his3-11,15 leu2-3,112 trp1-1 can1- 100 +pCEN/URA3/DED1*	This study
TBY 259	*MATαded1::KAN CST6:3XHA:HIS3 ade2-1 ura3-1 his3-11,15 leu2-3,112 trp1-1 can1- 100 +pCEN/LEU2/DED1*	This study
TBY 260	*MATαded1::KAN CST6:3XHA:HIS3 ade2-1 ura3-1 his3-11,15 leu2-3,112 trp1-1 can1- 100 +pCEN/LEU2/ded1-R310W*	This study
TBY 261	*MATαded1::KAN CST6:3XHA:HIS3 ade2-1 ura3-1 his3-11,15 leu2-3,112 trp1-1 can1- 100 +pCEN/LEU2/ded1-F316S*	This study
TBY 262	*MATαded1::KAN VMA5:3XHA:HIS3 ade2-1 ura3-1 his3-11,15 leu2-3,112 trp1-1 can1- 100 +pCEN/URA3/DED1*	This study
TBY 263	*MATαded1::KAN VMA5:3XHA:HIS3 ade2-1 ura3-1 his3-11,15 leu2-3,112 trp1-1 can1- 100 +pCEN/LEU2/DED1*	This study
TBY 264	*MATαded1::KAN VMA5:3XHA:HIS3 ade2-1 ura3-1 his3-11,15 leu2-3,112 trp1-1 can1- 100 +pCEN/LEU2/ded1-R310W*	This study
TBY 265	*MATαded1::KAN VMA5:3XHA:HIS3 ade2-1 ura3-1 his3-11,15 leu2-3,112 trp1-1 can1- 100 +pCEN/LEU2/ded1-F316S*	This study
TBY 266	*MATαded1::KAN EDE1:3XHA:HIS3 ade2-1 ura3-1 his3-11,15 leu2-3,112 trp1-1 can1- 100 +pCEN/URA3/DED1*	This study
TBY 267	*MATαded1::KAN EDE1:3XHA:HIS3 ade2-1 ura3-1 his3-11,15 leu2-3,112 trp1-1 can1- 100 +pCEN/LEU2/DED1*	This study
TBY 268	*MATαded1::KAN EDE1:3XHA:HIS3 ade2-1 ura3-1 his3-11,15 leu2-3,112 trp1-1 can1- 100 +pCEN/LEU2/ded1-R310W*	This study

TBY 269	*MATαded1::KAN EDE1:3XHA:HIS3 ade2-1 ura3-1 his3-11,15 leu2-3,112 trp1-1 can1- 100 +pCEN/LEU2/ded1-F316S*	This study

ded1-mam list (*MATα ded1::KAN ade2-1 ura3-1 his3-11,15 leu2-3,112 trp1-1 can1-100 +pCEN/LEU2/ded1-mam*):

**Strain name**	**Genotype**	**Source**
TBY53	*-RC285(pTB38)*	(35)
TBY54	*-F316S (pTB39)*	(35)
TBY55	*-P526L (pTB42)*	(35)
TBY56	*-R492H (pTB43)*	(35)
TBY57	*-T234M (pTB40)*	(35)
TBY58	*-R335C (pTB41)*	(35)
TBY59	*-S371F (pTB44)*	(35)
TBY60	*-A184P (pTB45)*	(35)
TBY61	*-R235K (pTB59)*	(35)
TBY62	*-D313H (pTB52)*	(35)
TBY63	*-M329R (pTB54)*	(35)
TBY64	*-L312F (pTB48)*	(35)
TBY65	*-D288V (pTB49)*	(35)
TBY66	*-G488V (pTB50)*	(35)
TBY67	*-R433C (pTB51)*	(35)
TBY68	*-T166A (pTB55)*	(35)
TBY69	*-V168L (pTB56)*	(35)
TBY70	*-R486H (pTB67)*	(35)
TBY71	*-L393D (pTB66)*	(35)
TBY72	*-G189V (pTB57)*	(35)
TBY73	*-T193A (pTB63)*	(35)
TBY74	*-G262V (pTB60)*	(35)
TBY75	*-L286V (pTB62)*	(35)
TBY76	*-M339I (pTB71)*	(35)
TBY77	*-V364L (pTB74)*	(35)
TBY78	*-T490M (pTB68)*	(35)
TBY79	*-R310W (pTB73)*	(35)
TBY80	*-G494R (pTB65)*	(35)
TBY81	*-E236Q (pTB64)*	(35)
TBY82	*-A352S (pTB76)*	(35)
TBY83	*-V454M (PTB78)*	(35)
TBY87	*-G462V (pTB88)*	(35)

TBY88	*-D326V (pTB89)*	(35)

Supplementary table S2: Plasmids used in this study

Plasmid Name	Description	Source
pRS315	*CEN/LEU2*	(51)
pSW3619	*CEN/LEU2/DED1*	(49)
pTB38	*CEN/LEU2/ded1-R285C*	(35)
pTB39	*CEN/LEU2/ded1-F316S*	(35)
pTB40	*CEN/LEU2/ded1-T234M*	(35)
pTB41	*CEN/LEU2/ded1-R335C*	(35)
pTB42	*CEN/LEU2/ded1-P526L*	(35)
pTB43	*CEN/LEU2/ded1-D464Y*	(35)
pTB44	*CEN/LEU2/ded1-S371F*	(35)
pTB45	*CEN/LEU2/ded1-A184P*	(35)
pTB46	*CEN/LEU2/ded1-R492H*	(35)
pTB47	*CEN/LEU2/ded1-G284E*	(35)
pTB48	*CEN/LEU2/ded1-L312F*	(35)
pTB49	*CEN/LEU2/ded1-D288V*	(35)
pTB50	*CEN/LEU2/ded1-G488A*	(35)
pTB51	*CEN/LEU2/ded1-R433C*	(35)
pTB52	*CEN/LEU2/ded1-D313H*	(35)
pTB53	*CEN/LEU2/ded1-G261V*	(35)
pTB54	*CEN/LEU2/ded1-M329R*	(35)
pTB55	*CEN/LEU2/ded1-T166A*	(35)
pTB56	*CEN/LEU2/ded1-V168L*	(35)
pTB57	*CEN/LEU2/ded1-G189V*	(35)
pTB59	*CEN/LEU2/ded1-R235K*	(35)
pTB60	*CEN/LEU2/ded1-G262V*	(35)
pTB61	*CEN/LEU2/ded1-H485Y*	(35)
pTB62	*CEN/LEU2/ded1-L286V*	(35)
pTB63	*CEN/LEU2/ded1-T193A*	(35)
pTB64	*CEN/LEU2/ded1-E236Q*	(35)
pTB65	*CEN/LEU2/ded1-G494R*	(35)
pTB66	*CEN/LEU2/ded1-L393!1*	(35)
pTB67	*CEN/LEU2/ded1-R486H*	(35)
pTB68	*CEN/LEU2/ded1-T490M*	(35)
pTB70	*CEN/LEU2/ded1-I374!1*	(35)

pTB71	*CEN/LEU2/ded1-M339I*	(35)
pTB72	*CEN/LEU2/ded1-T343P*	(35)
pTB73	*CEN/LEU2/ded1-R310W*	(35)
pTB74	*CEN/LEU2/ded1-V364L*	(35)
pTB76	*CEN/LEU2/ded1-A352S*	(35)
pTB77	*CEN/LEU2/ded1-A4591*	(35)
pTB78	*CEN/LEU2/ded1-V454M*	(35)
pTB88	*CEN/LEU2/ded1-G462V*	(35)
pTB89	*CEN/LEU2/ded1-D326V*	(35)
pTB90	*CEN/LEU2/ded1-Q240H*	(35)
pTB109	*CEN/LEU2/ded1-V176S*	(35)
pTB136	*CEN/LEU2/ded1-ΔCT*	(28)
ded1-120	*CEN/LEU2/ded1-120*	(13)
pFJZ342	*CEN/URA3/5'UTR-RPL41A+CAA(23)-LUC*	(21)
pFJZ623	*CEN/URA3/5'UTR-RPL41A+CAA(23)+distal-stem-loop(3.7)-LUC*	(21)

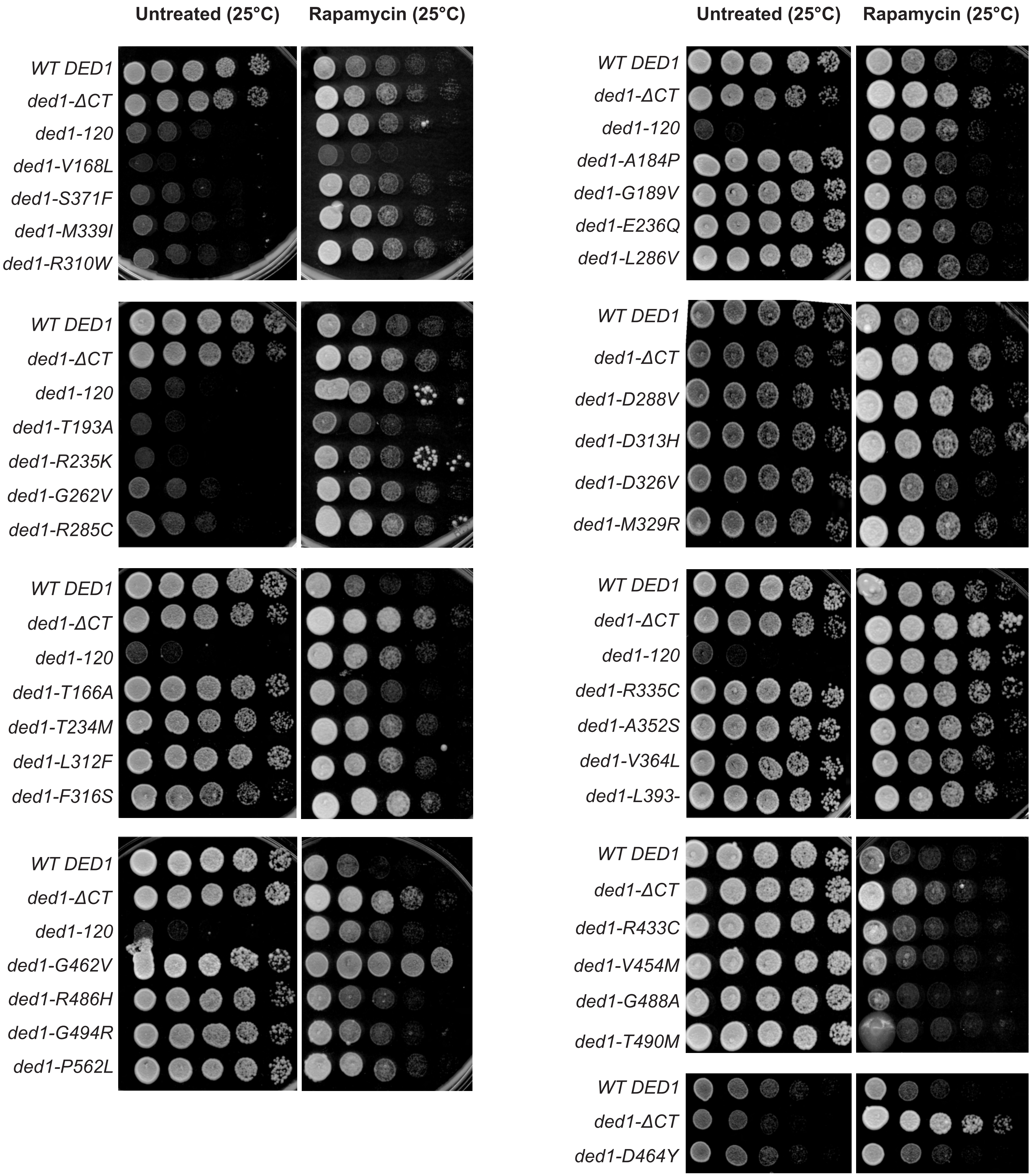

Figure S1: Growth and rapamycin-sensitivity of *DDX3X/DED1* medulloblastoma-associated mutations at 25°C. Wild-type *DED1* cells and the indicated *ded1-mam* mutants were serially diluted onto nutrient-rich agar in the presence or absence of rapamycin (200 ng/ml) and incubated at 25°C.

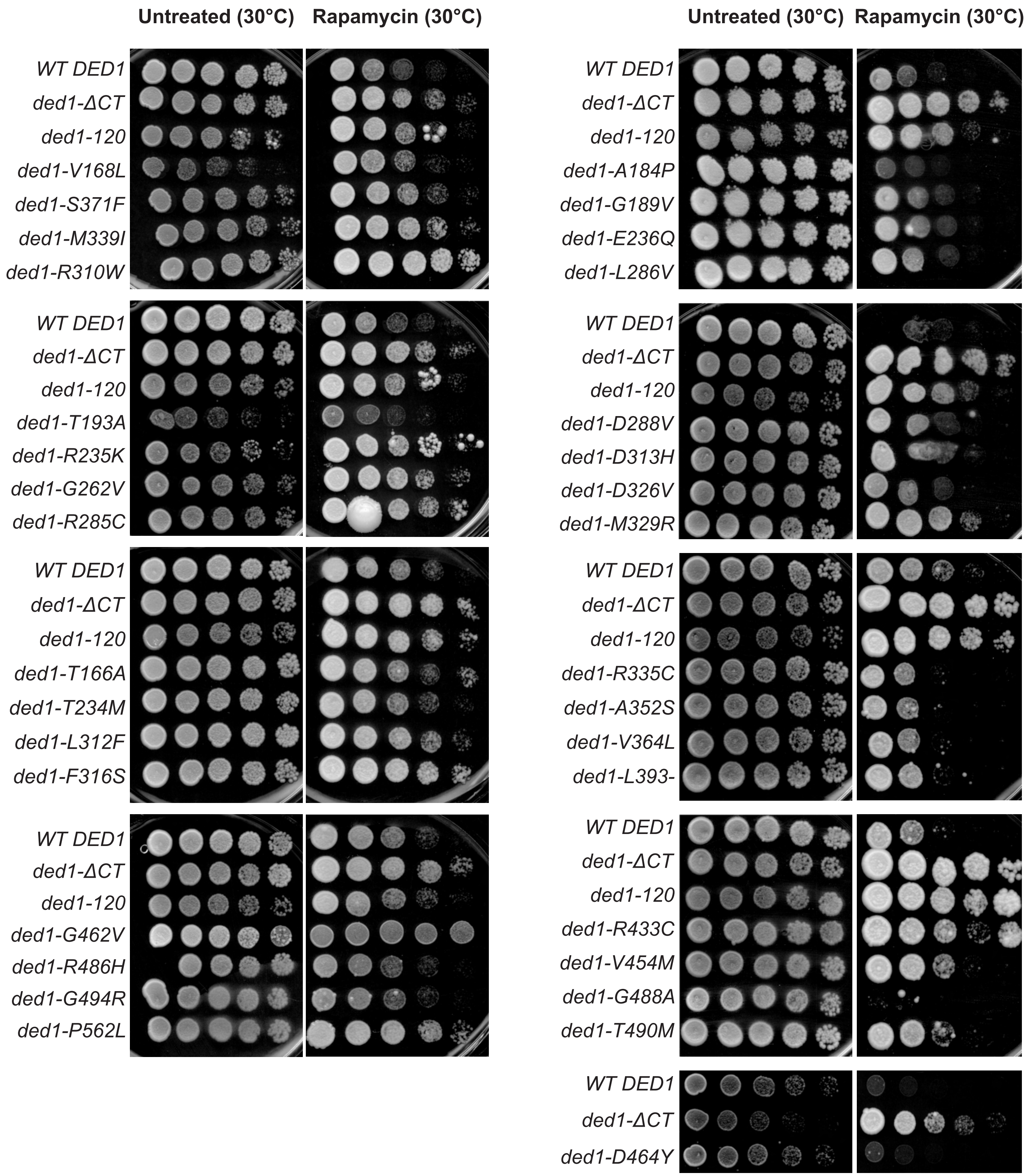

Figure S2: Growth and rapamycin-sensitivity of *DDX3X/DED1* medulloblastoma-associated mutations at 30°C. Wild-type *DED1* cells and the indicated *ded1-mam* mutants were serially diluted onto nutrient-rich agar in the presence or absence of rapamycin (200 ng/ml) and incubated at 30°C.

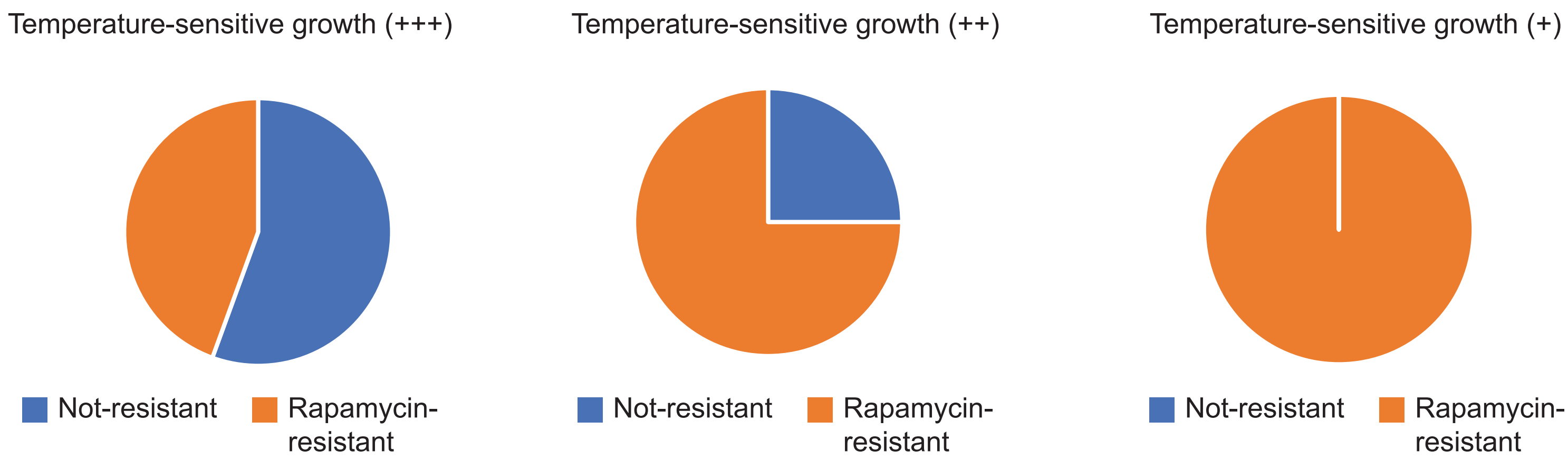

Figure S3: Correlation between growth phenotypes and rapamycin-resistance of *ded1-mam* cells. All non-lethal *ded1-mam* mutants were sorted into 3 categories based on their temperature sensitivity on rich media: normal growth at 25°C (+++), moderate growth (++), and poor growth (+). The percentage of mutants showing rapamycin-resistant growth in each category was then graphed. (+++) 8 out of 18 were rapamycin-resistant; (++) 6 of 8; for (+) 7 of 7.

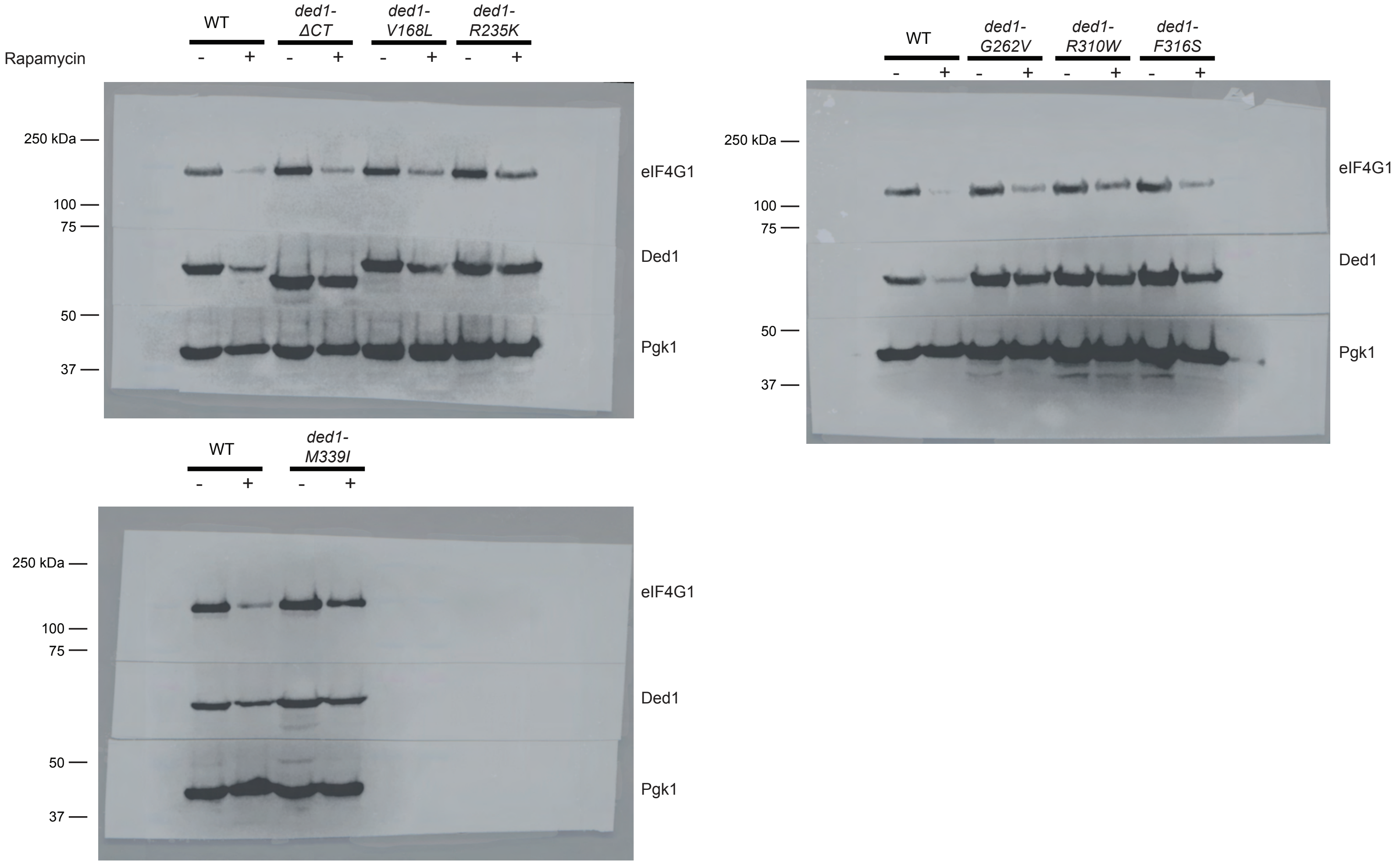

Figure S4: Original, uncropped western blots of Fig 3A. Increased levels of Ded1 and eIF4G1 are observed in the respective *ded1-mam* on rapamycin treatment.

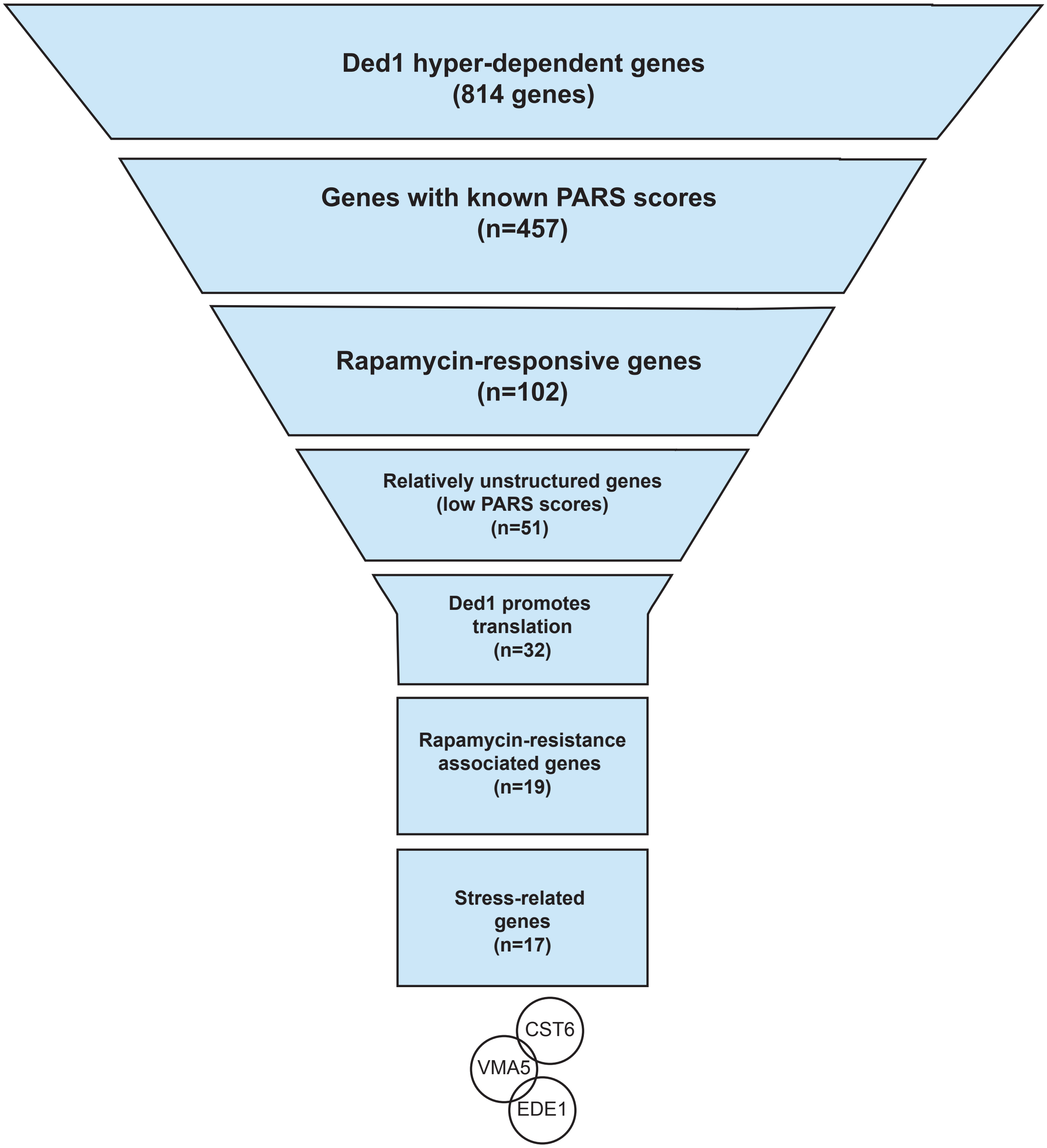

Figure S5: Selection of Ded1-dependent genes with relatively unstructured 5' UTRs. From a list of 814 Ded1-dependent genes, we selected 3 highly Ded1-dependent gene with relatively unstructured 5' UTRs that are rapamycin-resistance associated and stress-related.

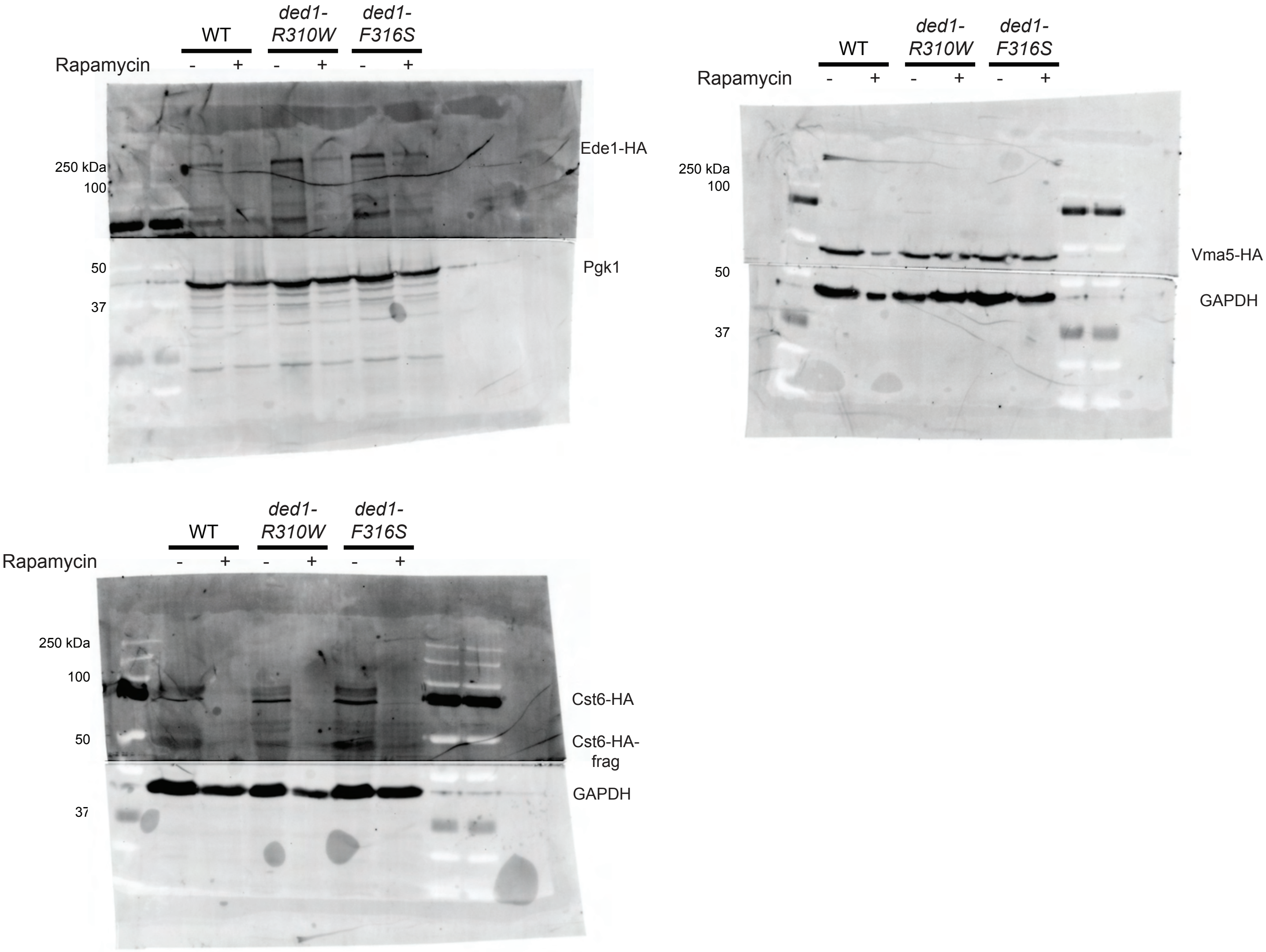

Figure S6: Original, uncropped western blots of Fig 5B-D. Increased levels of Ede1, Vma5 and Cst6 are observed in the respective *ded1-mam* on rapamycin treatment.
